# Expression profile of the matricellular protein periostin in paediatric inflammatory bowel disease

**DOI:** 10.1038/s41598-021-85096-7

**Published:** 2021-03-18

**Authors:** Tracy Coelho, Eva Sonnenberg-Riethmacher, Yifang Gao, Enrico Mossotto, Alisher Khojanazarov, Annie Griffin, Saida Mukanova, Aiymkul Ashimkhanova, Rachel Haggarty, Anton Borissenko, James J. Ashton, Imogen S. Stafford, Akshay Batra, Nadeem A. Afzal, Michael P. Stanton, Bhumita Vadgama, Kapura Adrisova, Robert M. Beattie, Anthony P. Williams, Sarah Ennis, Dieter Riethmacher

**Affiliations:** 1 Human Genetics and Genomic Medicine, University of Southampton, Southampton, UK; 2 Department of Biomedical Sciences, Nazarbayev University School of Medicine, Nur-Sultan, Republic of Kazakhstan; 3 Department of Paediatric Gastroenterology, University Hospital Southampton, Southampton, UK; 4 Academic Unit of Human Development and Health, University of Southampton, Southampton, UK; 5 Cancer Sciences Division, Faculty of Medicine, University Hospital Southampton, Southampton, UK; 6 Southampton Biomedical Research Centre, NIHR, University Hospital Southampton, Southampton, UK; 7 Department of Paediatric Surgery, University Hospital Southampton, Southampton, UK; 8 Department of Pathology, University Hospital Southampton, Southampton, UK; 9 National Research Center for Maternal and Child Health, Nur-Sultan, Republic of Kazakhstan; 10 Organ Transplantation Center, The First Affiliated Hospital, Sun Yatsen University, Guangzhou, China

**Keywords:** Chronic inflammation, Diagnostic markers, Crohn's disease, Ulcerative colitis, Gene expression

## Abstract

The precise role of periostin, an extra-cellular matrix protein, in inflammatory bowel disease (IBD) is unclear. Here, we investigated periostin in paediatric IBD including its relationship with disease activity, clinical outcomes, genomic variation and expression in the colonic tissue. Plasma periostin was analysed using ELISA in 144 paediatric patients and 38 controls. Plasma levels were assessed against validated disease activity indices in IBD and clinical outcomes. An immuno-fluorescence for periostin and detailed isoform-expression analysis in the colonic tissue was performed in 23 individuals. We integrated a whole-gene based burden metric ‘GenePy’ to assess the impact of variation in *POSTN* and 23 other genes functionally connected to periostin. We found that plasma periostin levels were significantly increased during remission compared to active Crohn’s disease. The immuno-fluorescence analysis demonstrated enhanced peri-cryptal ring patterns in patients compared to controls, present throughout inflamed, as well as macroscopically non-inflamed colonic tissue. Interestingly, the pattern of isoforms remained unchanged during bowel inflammation compared to healthy controls. In addition to its role during the inflammatory processes in IBD, periostin may have an additional prominent role in mucosal repair. Additional studies will be necessary to understand its role in the pathogenesis, repair and fibrosis in IBD.

## Introduction

Tissue repair and remodelling is a key component of the adaptations which occur following injury or inflammation^1^. The matricellular protein periostin, encoded in humans by the *POSTN* gene in the long arm of chromosome 13, is a key player in tissue repair and remodelling^2^. The multi-faceted role of this intriguing molecule has been described in a number of immune-mediated inflammatory conditions^2,3^. Its role in cancer development and progression has also been well-described^4–6^. Recent studies have implicated its role in inflammatory bowel disease (IBD)^7,8^, however the precise mechanisms through which it influences the immune signalling pathways in IBD remains unclear, with varying results observed in patients and animal models^7–9^.

The two main categories of IBD include Crohn’s disease (CD) and ulcerative colitis (UC), with an additional category IBD-unclassified (IBDU), where a clear distinction between the two main categories is not possible due to overlapping features. CD is characterised by inflammation of any part of the gastro-intestinal tract (GIT) from the oral cavity to the anus, whereas UC typically includes inflammation of the colon in a contiguous manner upstream beginning in the rectum^10^. IBD is a chronic inflammatory condition associated with a considerable morbidity and a significantly reduced quality of life^11^.

IBD is a complex immune-mediated polygenic disease, in which the dysregulation of key immune signalling pathways has been identified as an important operating mechanism^10,12^. Aberrant function of the NF-κB pathway, a pivotal mediator of inflammatory immune responses, has long been considered to be a key factor in the development of IBD^10^. As periostin is functionally connected to the NF-κB pathway, particularly in inflammatory states, investigating the functionality of this rather obscure protein will be an important step forward for a better understanding of IBD^7,13,14^.

A variety of isoforms of periostin (which differ in the composition of exons 16 to 23) have been described^15,16^. The expression of these different isoforms depends on the tissue type as well the specific circumstances such as inflammation, remodelling or repair. Given the involvement of this pleiotropic protein in a number of distinct functions, it is likely that the different isoforms contribute to the different observed functions of periostin.

Several studies suggest that periostin is a key player in the development of tissue fibrosis through mechanisms involving complex interactions between the extra-cellular matrix (ECM) and cell-surface αv-integrin receptors, localisation of fibrogenic inflammatory mediators and regulation of mesenchymal growth factors^17–19^. Dysregulation of tissue repair mechanisms following inflammation can result in excessive fibrosis with the formation of scar tissue^2^. Scarring of the bowel wall leading to stricture formation is a significant cause of morbidity in IBD, necessitating surgical interventions and impacting adversely on the quality of life^20^. Previous studies have demonstrated altered serum levels of periostin in conditions associated with inflammation and fibrosis^21–24^. Although emerging evidence suggests increased expression of periostin in the mucosal tissue of patients with ulcerative colitis (UC)^7,8^, its’ relationship with disease activity or fibrosis has not been investigated^25^.

Periostin has emerged as a potential biomarker of disease, with altered serum levels observed in various conditions associated with inflammation, allergy and fibrosis^21,26–29^. However, in the paediatric age group, studies have demonstrated a variable trend in the serum levels of the protein with very high levels during infancy, followed by a decline until age seven, and thereafter an increasing trend until fifteen years of age, in keeping with active bone metabolism^26,30,31^. Hence, this is a major drawback in using this protein as a biomarker of disease in childhood. Overall, the serum levels of periostin have been observed to significantly higher in children compared to adults^26,30,31^.

Our study explores the feasibility of using periostin to assess disease activity in paediatric inflammatory bowel disease (pIBD), and its role in inflammation and repair.The study assesses plasma periostin levels in pIBD, investigating its relationship with disease activity, surgical outcomes and genomic variation. Furthermore, the study investigates the expression of periostin in the GI tissue in healthy and diseased bowel.

## Methods

The subjects described in this study included paediatric IBD patients, paediatric controls and adult controls from a UK cohort. The study also included paediatric patients from a Kazakh cohort. Informed consent was obtained from all participants of this study.

### UK cohort

This cohort included individuals recruited as part of a prospective observational study ‘Genetics of IBD- Southampton’, a registered portfolio member of the NIHR (National Institute for Health Research) Clinical Research Network (CLRN ID 11158). Individuals included in this study were recruited from October 2010 to December 2016 from the ‘Genetics of IBD-Southampton’ cohort and their biological samples collected during that period. All patients were actively followed up through the course of their disease and treatment. The three groups included in the UK cohort were as follows.

#### Paediatric patients

Patients under the age of 18 years referred to the regional paediatric gastroenterology service as suspected IBD were identified and recruited to the ‘Genetics of IBD’ study. The IBD working group- Porto criteria were used to establish a diagnosis of IBD^32^.

#### Paediatric controls

These participants included children, who were referred with gastro-intestinal symptoms but did not have a diagnosis of IBD, had normal endoscopic/histological examination and remained well for a minimum period of six months of follow-up.

#### Adult controls

Control subjects for plasma assessment also included healthy adult individuals over the age of eighteen years recruited as part of the control arm of the ‘Genetics of IBD’study.

### Kazakh cohort

Paediatric patients with an established diagnosis of IBD were also recruited from the National Research Center for Maternal and Child Health, Nazarbayev University, Republic of Kazakhstan. Only gastro-intestinal (GI) tissue specimens were utilised for periostin analysis from the Kazakh cohort. The GI specimens were obtained during routine endoscopy performed on the GI unit for clinical reasons.

#### Measuring disease activity

At the time of specimen collection, disease activity was recorded using validated assessment tools, Paediatric Crohn’s Disease Activity Index (PCDAI) and Paediatric Ulcerative Colitis Activity Index (PUCAI) for CD and UC respectively^25,33,34^, with a disease score < 10 indicating clinical remission in both conditions. The scoring indices for defining active disease were as follows: PUCAI (scores range from 0 to 85; remission or inactive disease < 10, mild disease 10–34, moderate 35–64, severe > 65) for UC and PCDAI (scores range from 0–100; remission or inactive disease < 10, mild 10–29, moderate-severe > 30) for CD. See Table S1A,B in the supplementary for further details.

#### Plasma analysis

Plasma specimens were collected from patients and controls at the point of recruitment to the study. Of the 142 patients who had their plasma samples analysed for periostin, eleven patients had their samples taken at diagnosis before commencement of any treatment. The remaining patients already had an established diagnosis of IBD at the time of plasma sample procurement. The disease activity scores were recorded at the time of plasma collection in order to group patients into those with active disease and those in clinical remission. All the eleven patients who had their samples taken at diagnosis had active disease. These eleven patients also had a GI specimen taken during endoscopy for immunofluorescence alongside their plasma collection. Plasma levels of periostin were measured using ELISA technique according to manufacturer’s instructions (Human Periostin/OSF2 DuoSet ELISA, R & D systems). See online supplementary- SM Section 1.

#### Immuno-fluorescence and isoform analysis on GI specimen

Mucosal biopsies taken during routine lower GI endoscopy were assessed for periostin expression in twenty-three individuals, two biopsies each from apparently normal looking areas of the colon and two from the mucosa with macroscopic features of inflammation (where appropriate). GI biopsy specimens were available for immunofluorescence and isoform expression analysis in all of the twenty-three individuals. In the UK patient cohort, GI biopsy specimens were obtained at diagnosis prior to commencement of treatment for IBD. A whole thickness colonic specimen was obtained from one individual with an established diagnosis of CD. This patient had shown poor response to medical treatment and underwent a right hemi-colectomy for severe ileo-caecal stricturing disease. In the Kazakhstan cohort, GI biopsy specimens were also obtained during routine GI endoscopy. However, all patients had an established diagnosis of IBD and were on treatment for their disease. Detailed steps of the immuno-staining procedure and periostin isoform analysis are included in the online supplementary material- SM sections 2 & 3.

#### Genomic methods

Whole exome sequencing (WES) was performed using peripheral blood samples or from saliva collected at the time of recruitment to the study. WES data were analysed using a whole gene-based pathogenicity score, ‘GenePy’ as previously described^35^. This whole gene-based score was applied across a panel of prioritised genes which were selected for their functional relevance to periostin. For the selection of genes, an electronic search was conducted through ‘PathCards’, an online database of human biological pathways^36^. The combined GenePy scores for the selected genes were compared between groups of patients to determine the impact of genetic variants, which could potentially influence periostin levels and the need to undergo surgical interventions. Further details on DNA extraction and WES data analysis is included in the online supplementary- SM sections. 4–6.

#### Patients treated with surgical interventions

All patients undergoing plasma analysis had their electronic medical records analysed to check if they ever needed a surgical intervention for the treatment of their disease during the period of follow up. The indications for surgical or endoscopic interventions included strictures/fibro-stenosing disease, as well as severe luminal disease refractory to standard medical treatments. Surgical treatments included dilatation of strictures, stricturoplasty and resection of the diseased portion of the bowel including colectomy. Given the predominant role of periostin in tissue repair and remodelling, these surgical interventions were used as proxy markers for fibrosis/scarring of the bowel and dysregulated mucosal repair^25^. Periostin parameters were compared between patients needing surgical treatments (including endoscopic therapeutic procedures) and those who did not need these interventions for their disease.

#### Statistical analysis

Descriptive statistics were presented with numbers and percentages where applicable, and numerical data as mean with 95% confidence intervals or median with interquartile ranges (where appropriate). Statistical analysis was performed using Prism software (GraphPad- V7). The significance of comparisons for quantitative parameters was determined using 2-tailed t-tests and regression analysis. Differences with *p* < 0.05 were considered statistically significant.

#### Ethical considerations

The study protocol was approved by the South Central- Hampshire B Research Ethics Committee (09/H0504/125) in February 2010. A written consent was obtained from all study participants, including their parents/legal guardians where appropriate. Recruitment of individuals from the Kazakh cohort to the study was ethically approved by the ethical board from the National Research Center for Maternal and Child Health affiliated to the Nazarbayev University Medical Center (UMC) and Nazarbayev University Institutional Research Ethics Committee (IREC), Nur-Sultan, Republic of Kazakhstan. The study protocol conforms to the ethical guidelines of the 1975 declaration of Helsinki.

## Results

### Cohort characteristics for periostin plasma levels analysis

The patient cohort (n = 144) included 65% males (n = 93) and 35% females (n = 51), median age = 14 years (range 4–17 years), 71% (n = 102) accounting for CD and 29% of the patients with UC (n = 42). Observational follow-up since the first diagnosis of IBD was for a median period of 32 months. For baseline patient and control characteristics see Table 1. Tables S2A,B in the supplementary include details on the phenotypic description of the disease in individual patients as per the Paris classification^37^ for IBD and the disease activity state where available.

**Table 1:** Cohort characteristics undergoing plasma periostin analysis.

	Male (%)	Female (%)	Total	Median age in years (age range)
Crohn's disease	71 (70)	31 (30)	102	14 (5–17)
Ulcerative colitis	22 (52)	20 (48)	42	14.4 (4–16)
Paediatric controls	9 (69)	4 (31)	13	13.3 (4–16)
Adult controls	10 (40)	15 (60)	25	24 (19–40)

### Disease activity scores for plasma analysis

At the time of plasma sample collection, PUCAI scores were available in all patients with a score indicative of active disease (≥ 10) recorded in 21 patients (50%). The median PUCAI score in patients with active UC was 22.5 (IQR 16.25–43.75). Generation of PCDAI scores was possible in only 68% (n = 69) of the CD patients. Of these, active Crohn’s disease with a PCDAI score ≥ 10 was recorded in 48 individuals (median score 25; IQR 20–35).

### Comparison of peripheral blood periostin levels

**Figure 1:**
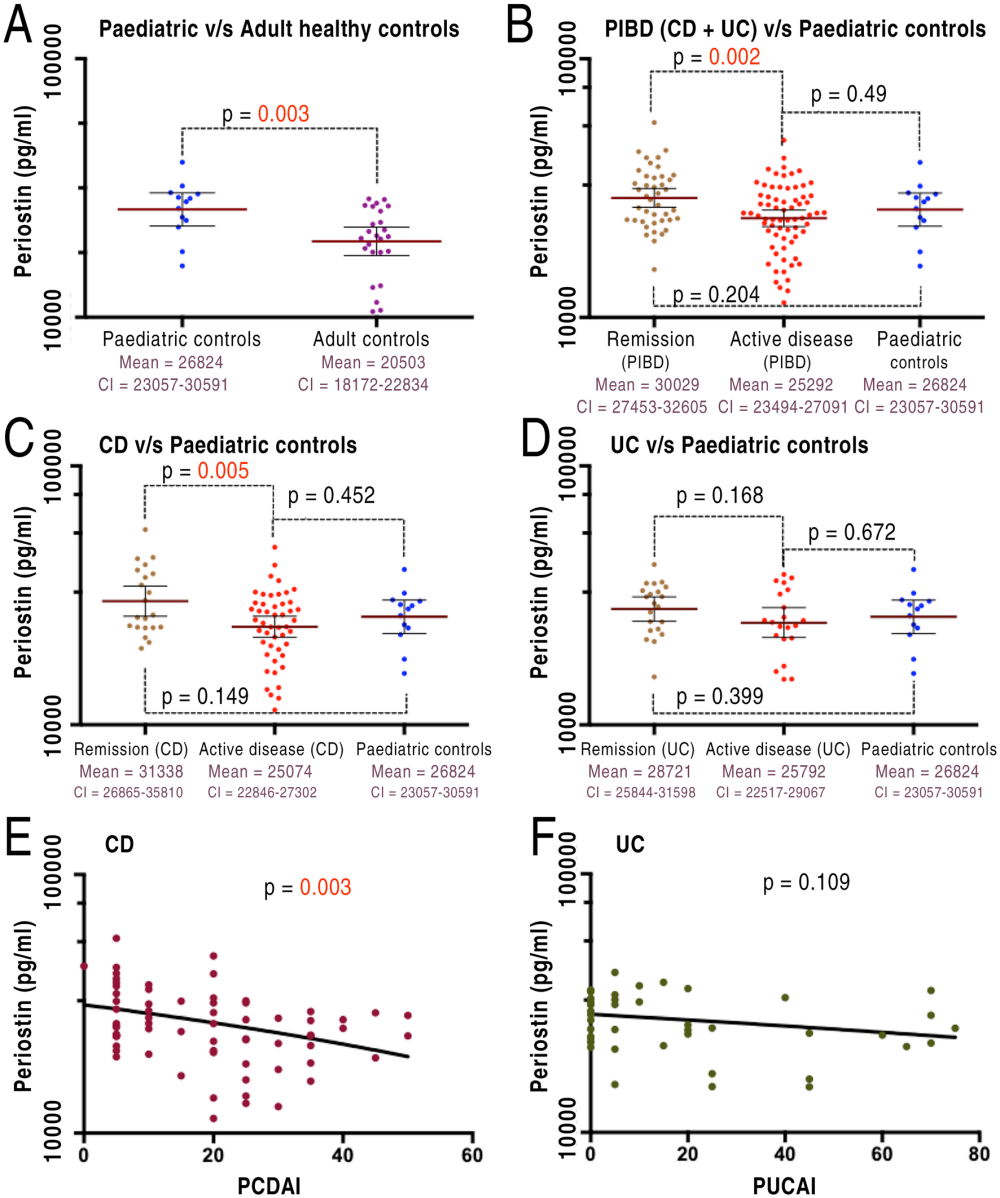
Plasma periostin levels against disease activity. (**A**) Significantly lower levels in adult controls in comparison to paediatric controls (*p* = 0.003). (**B**) Higher levels during remission (combined for both CD & UC) than active disease (*p* = 0.002). (**C**) Statistically significant differences in periostin levels during remission driven by CD; remission (31,338 ± 2144) compared to active disease (25,074 ± 1108); *p* = 0.005. (**D**) Patients with UC show no significant differences during remission compared to active disease. (**E**) An inverse relationship between periostin levels and PCDAI scores (**F**) No relationship between periostin and disease activity in UC. This figure was, as also mentioned in the methods/stats section, generated through PRISM https://www.graphpad.com/scientific-software/prism/ Version 7. Abbreviations: CD- Crohn’s disease; PCDAI- paediatric Crohn’s disease activity index; PUCAI- Paediatric ulcerative colitis activity index; pIBD- paediatric inflammatory bowel disease (includes both Crohn’s disease and ulcerative colitis); UC- ulcerative colitis.

As shown in Fig. 1, significantly higher levels of plasma periostin were observed in paediatric controls (median age = 13.3, range: 4–16) compared to adult controls (median age = 24, range: 19–40). Due to the significant differences between the adult and paediatric controls, measurements from adult controls were excluded from further comparative analysis. Crohn’s disease patients in remission showed significantly higher levels compared to those with active disease (Fig. 1C), with a linear regression plot demonstrating an inverse association between disease activity scores and periostin levels (r^2^ = 0.1196, *p* = 0.003) (Fig. 1E). However, no relationship was observed between the disease activity scores and periostin levels in patients with UC (Fig. 1F). Plasma levels were also not significantly different between patients and paediatric controls. Also, there was no significant association between the age of patients and periostin levels (Fig. S1 in the supplementary).

### Patients treated with surgical interventions

The group of patients with CD who underwent surgical interventions for the treatment of their disease (n = 17) showed no significant differences in their plasma levels compared to non-surgical patients (n = 85). Likewise, periostin levels did not differ significantly in UC patients who needed surgery (n = 6) for the treatment of their disease compared to the non-surgical group (n = 36). (See Fig. S2 in the supplementary. Details of the surgical procedures are described in Table S3 in the supplementary.

### Immuno-fluorescence on colonic biopsies

Immuno-fluorescence for periostin was performed on colonic tissue in seventeen individuals from the UK cohort (median age 14 years) and six individuals from the Kazakh cohort (median age 14 years). In the UK cohort, ten patients had CD, two patients had UC and five individuals were non-IBD paediatric controls. All specimens except one (individual Soton-CD1 from the UK cohort), were collected at diagnosis before commencement of any treatment. In the individual Soton-CD1, whole thickness bowel tissue was obtained following a right hemicolectomy for an ileo-caecal stricturing CD and immuno-fluorescence was performed on the whole-thickness tissue. In the remaining individuals, mucosal biopsies taken during endoscopy were utilised for immuno-fluorescence. The Kazakh patient cohort for the immuno-fluorescence analysis included two individuals with CD and four with UC. Disease activity scores were not available in the Kazakh cohort, however, all six patients had clinically active disease at the time of sample procurement (see Table 2).

**Table 2:** Immuno-fluorescence staining for periostin: patients and controls. Immuno-fluorescence staining for periostin was performed in 23 individuals including 5 non-IBD controls. PUCAI or PCDAI are disease activity scores for UC and CD respectively at the time of tissue procurement. AZA—azathioprine. Disease location/s as per Paris classification for Crohn’s disease: L2 (colonic), L3 (ileo-colonic) & L4 (upper GI disease; L4a- proximal to the ligament of Treitz, L4b- distal to the ligament of Treitz); disease location E4 for UC indicates pancolitis. (Abbreviations: N/Av—Not available, N/Ap—Not applicable).

	Cohort	ID Number	Gender	Age (years)	Disease/ Control	GI Tissue	Tissue status on macroscopy	Tissue obtained at diagnosis	Disease Scores	Disease status	Paris Classification (disease location)	Drugs
		**Crohn's Disease**										
1	UK	Soton-CD-1	M	14	CD	Surgical	Scarred tissue	No	10	Active	L3	AZA
2	UK	Soton-CD-2	M	16	CD	Mucosal	Normal & inflmd	Yes	30	Active	L3	None
3	UK	Soton-CD-3	F	14	CD	Mucosal	All inflammed	Yes	40	Active	L3L4a	None
4	UK	Soton-CD-4	M	11	CD	Mucosal	All inflammed	Yes	25	Active	L3L4a	None
5	UK	Soton-CD-5	M	15	CD	Mucosal	Normal & inflmd	Yes	30	Active	L3	None
6	UK	Soton-CD-6	M	12	CD	Mucosal	All inflammed	Yes	25	Active	L2L4a	None
7	UK	Soton-CD-7	M	13	CD	Mucosal	Normal & inflmd	Yes	25	Active	L3	None
8	UK	Soton-CD-8	M	16	CD	Mucosal	Normal & inflmd	Yes	35	Active	L3	None
9	UK	Soton-CD-9	M	12	CD	Mucosal	Normal & inflmd	Yes	35	Active	L3L4a	None
10	UK	Soton-CD-10	M	13	CD	Mucosal	Normal	Yes	20	Active	L3	None
11	Kazakh	Kaz-CD-1	M	16	CD	Mucosal	N/Av	No	N/Av	Active	N/Av	None
12	Kazakh	Kaz-CD-2	F	16	CD	Mucosal	N/Av	No	N/Av	Active	N/Av	None
		**Ulcerative Colitis**										
1	UK	Soton-UC-1	M	14	UC	Mucosal	All inflamd	Yes	75	Active	E4	None
2	UK	Soton-UC-2	F	15	UC	Mucosal	Normal & inflmd	Yes	70	Active	E4	None
3	Kazakh	Kaz-UC-1	F	12	UC	Mucosal	N/Av	No	N/Av	Active	N/Av	AZA
4	Kazakh	Kaz-UC-2	F	14	UC	Mucosal	N/Av	No	N/Av	Active	N/Av	Infliximab
5	Kazakh	Kaz-UC-3	F	8	UC	Mucosal	N/Av	No	N/Av	Active	N/Av	Steroids
6	Kazakh	Kaz-UC-4	F	8	UC	Mucosal	N/Av	No	N/Av	Active	N/Av	Infliximab
		**Controls**										
1	UK	Soton-C- 1	M	14	Control	Mucosal	Normal	Yes	N/Ap	N/Ap	N/Ap	None
2	UK	Soton-C- 2	M	10	Control	Mucosal	Normal	Yes	N/Ap	N/Ap	N/Ap	None
3	UK	Soton-C- 3	F	11	Control	Mucosal	Normal	Yes	N/Ap	N/Ap	N/Ap	None
4	UK	Soton-C- 4	M	14	Control	Mucosal	Normal	Yes	N/Ap	N/Ap	N/Ap	None
5	UK	Soton-C- 5	F	13	Control	Mucosal	Normal	Yes	N/Ap	N/Ap	N/Ap	None

**Figure 2:**
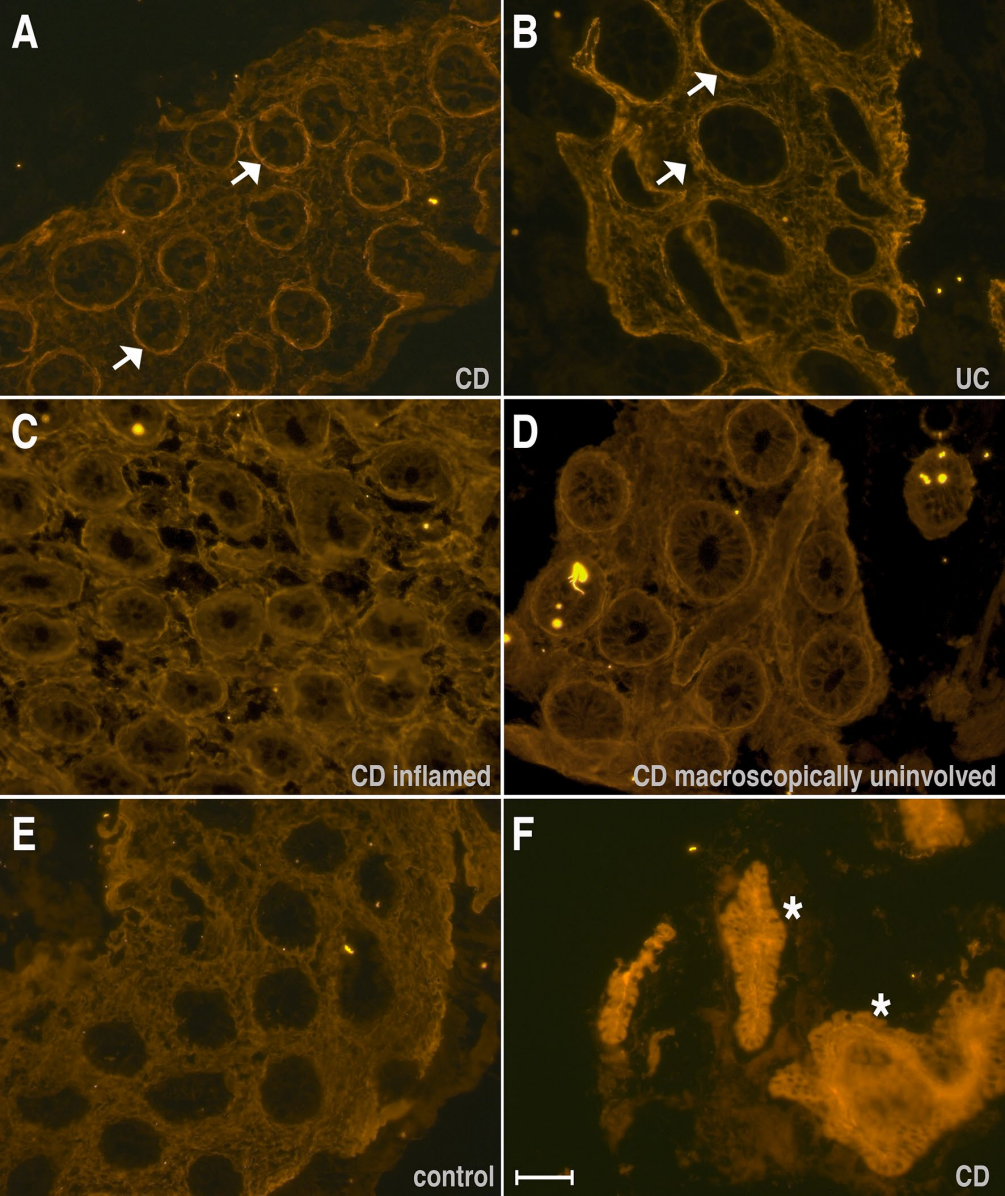
Immuno-fluorescence staining for periostin in colonic tissue. The figure shows representative immuno-fluorescence for periostin on the colonic tissue obtained from controls and IBD patients in the active state. Note the distinctly localised peri-cryptal rings that can be seen in CD (**A**, **C**, **D**) and UC (**B**) patients but not in the healthy control tissue (**E**). Interestingly, the peri-cryptal rings can be seen also in the macroscopically unaffected but histologically inflamed colonic mucosa in CD patients (**D**). These high intense areas appear more pronounced in CD patients compared to UC patients. Also note the very intense and diffuse staining of the epithelium and stromal tissue in the surgically resected bowel segment of a CD patient (**F**). Scale bar is 50 µm.

Immuno-fluorescence staining for periostin showed a localisation pattern around the epithelium lining the crypts with a characteristic ‘peri-cryptal ring’ appearance in the mucosal tissue of IBD patients in the active state, with CD patients in general showing a slightly more intense staining compared to UC patients (see Fig. 2A,B). Whilst an intense peri-cryptal localisation was observed in the inflamed areas of the mucosa of CD patients (see Fig. 2C), the localisation pattern was apparent even in biopsies taken from macroscopically spared areas of the colon (see Fig. 2D). Compared to patients, there was a low ubiquitous stromal distribution observed in controls (see Fig. 2E). In the surgically resected specimen with a stricture and histologically proven fibrosis, a very intense and diffuse staining of the epithelium and stromal tissue was observed (see Fig. 2F).

### Periostin isoforms expressed in paediatric IBD patients and controls

**Figure 3:**
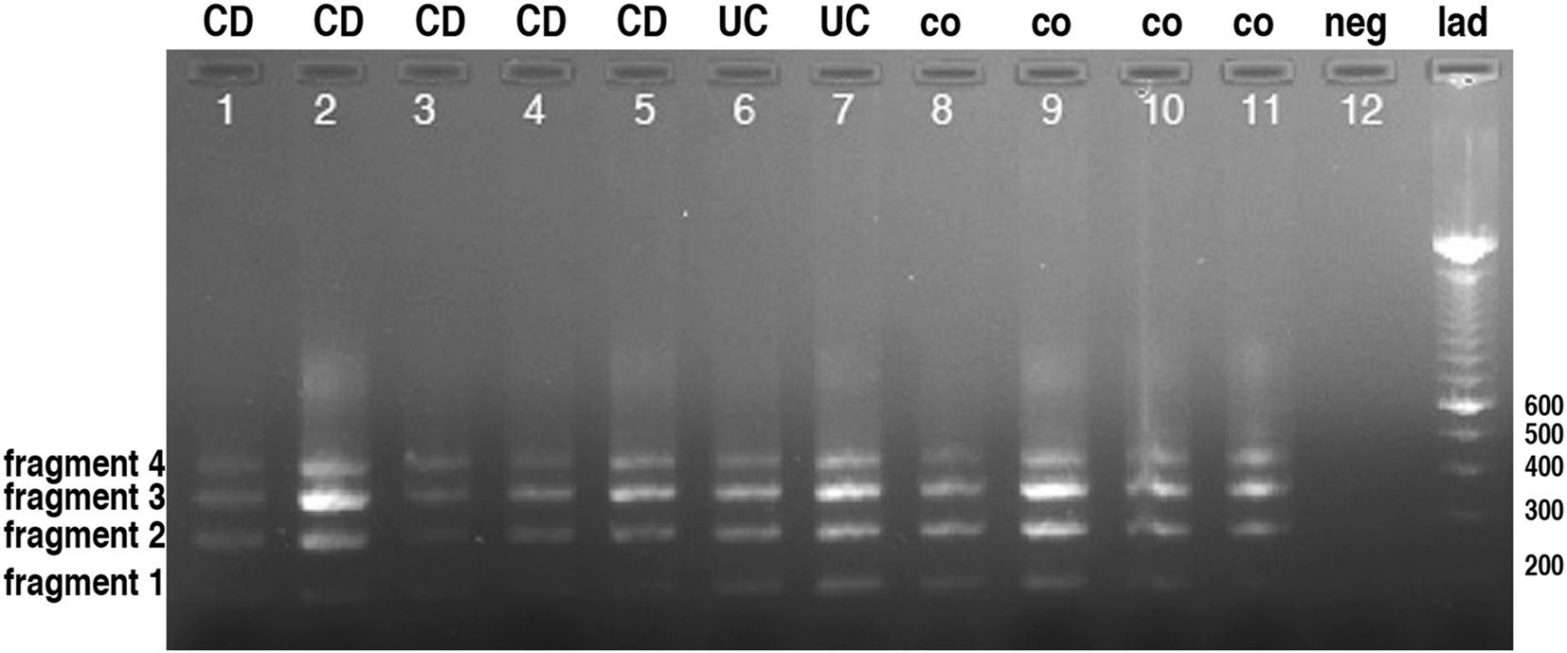
Periostin isoforms in the colonic tissue. The gel shows the isoforms present in tissue biopsies of CD patients (lanes 1–5), UC patient (lanes 6–7) and non-IBD controls (lanes 8–11). Lane 12 shows the negative control. Please note that there are minor fluctuations between the intensities of the different fragments that do not show any correlation between diseases CD (1–5) or UC (6–7), or controls (8–11). Fragment 1 (188 bp) is just below the 200 bp marker while fragment 4 (452 bp) is between 400 and 500 bp.

Periostin isoform specific PCR was performed on cDNA derived from UC and CD patients as well as non-IBD controls. As stated in the supplementary methods section, primers in exon 16 and 23 were used for the splice variant PCR reaction. There is no alternative splicing in exons 1–16 and only exons 17–22 show alternative splicing^16,38^. In all cases, four specific fragments could be observed (see Fig. 3). The pattern was identical in patients as well the non-IBD controls indicating that even though there are distinct localisation patterns of periostin in IBD compared to non-IBD-controls (see Fig. 2), there seems to be no shift in the expression of isoforms.

**Figure 4:**
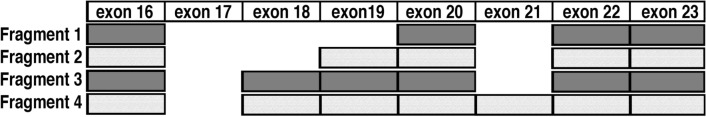
Sequencing of the periostin isoforms. Based on sequencing the exon usage of the four periostin isoforms isolated from the GI tissue in patients and healthy controls is shown. No alternative splicing occurs in exons 1–16 and the last exon 23 with the stop codon is also present in all variants. Fragment 1 contains exons 16, 20, 22 and 23 and is exactly 188 bp long, while fragment 2 contains additionally exon 19 and is 278 bp long. Fragment 3 additionally has exon 18 present and is 368 bp long and fragment 4 contains also exon 21 and is 452 bp long. Interestingly, we did not identify any trace of the full-length fragment that also retains exon 21 and would be expected to be 536 bp in length.

Polymerase chain reactions were used to clone all present fragments. We were not able to sub-clone any additional variants other than the four that were clearly visible in the gel analysis. Fragments one to four were sequenced to verify the exact exon composition of all four different isoforms (see Fig. 4).

### Sequencing data analysis

**Figure 5:**
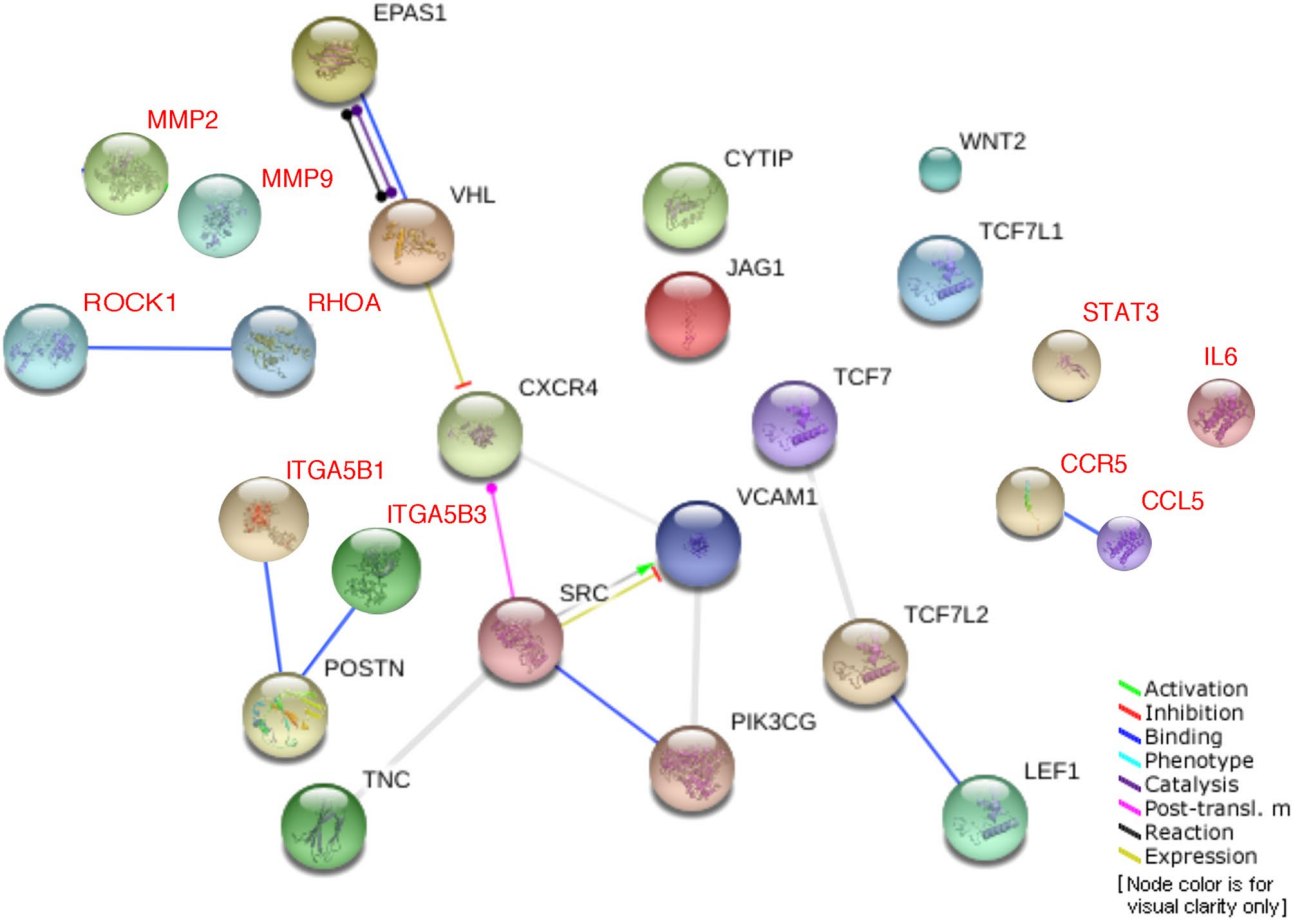
Network of genes for periostin. Screenshot from PathCards displaying seventeen SuperPath genes (black text) functionally connected to periostin and ten additional genes, known to be related to or influencing periostin signalling (red text). The protein nodes and the inter-connections within the retrieved network are automatically coloured for visual clarity and also to indicate the type of functional connectivity. The genes displayed in this figure are: C-X-C Motif Chemokine Receptor 4 (CXCR4); Cytohesin 1 Interacting Protein (CYTIP); Endothelial PAS Domain Protein 1 (EPAS1); Jagged Canonical Notch Ligand 1 (JAG1); Lymphoid Enhancer Binding Factor 1 (LEF1); Periostin (POSTN); Phosphatidylinositol-4,5-Bisphosphate 3-Kinase Catalytic Subunit Gamma (PIK3CG); SRC Proto-Oncogene, Non-Receptor Tyrosine Kinase (SRC); Tenascin C (TNC); Transcription Factor 7 (TCF7); Transcription Factor 7 Like 1 (TCF7L1); Transcription Factor 7 Like 2 (TCF7L2); Vascular Cell Adhesion Molecule 1 (VCAM1); Von Hippel-Lindau Tumor Suppressor (VHL); Wnt Family Member 2 (WNT2), Rho-associated protein kinase 1 (ROCK1), Ras Homolog Family Member A (RHOA), C–C chemokine receptor type 5 (CCR5), C–C Motif Chemokine Ligand 5 (CCL5), Matrix metalloproteinase-2 (MMP2), Matrix metalloproteinase-9 (MMP9) Signal transducer and activator of transcription 3 (STAT3), Interleukin 6 (IL-6) Integrin alpha V beta 1 & 3 (ITGA5B1&3). The screenshot was taken from the following url in April 2018. https://pathcards.genecards.org/Card/amplification_and_expansion_of_oncogenic_pathways_as_metastatic_traits?queryString=POSTN The screenshot database is based on a publication from Belinky et al.^36^ and was modified based on results in recent publications^6,39^.

Exome sequencing data were available in 78% (n = 112) of the patient cohort. A list of twenty-seven genes were prioritised for investigating deleterious mutations in the periostin-linked genes. These genes were selected through an electronic search on ‘Pathcards’ (online database of human biological pathways)^36^ and expert review/consensus within our research group^6,39^. See Fig. 5 for the selected network of genes, functionally connected to periostin. Mutational analysis in the selected gene panel was conducted using whole gene pathogenicity score ‘GenePy’^35^, as previously described. For the final analysis, the gene list was narrowed down to twenty-four as GenePy scores were available only for twenty-four of the twenty-seven selected genes in the cohort. Regression analysis across the twenty-four prioritised genes using GenePy scores per patient did not show any association between the plasma periostin levels and the mutational burden in the selected genes (Fig. S3 in the supplementary). Regression analysis was conducted individually for all of the 24 genes as well as after combining the GenePy scores for all these genes. Furthermore, using unpaired t-tests of GenePy scores, no significant differences in the mutational burden were observed between patients who underwent surgical interventions compared to those who did not need surgical interventions. (see Fig. S3 in the supplementary). Tables S4-5 in the supplementary include details of the GenePy scores for the selected genes in the CD and UC cohort respectively.

## Discussion

In this study, we observe an inverse association between circulating periostin and disease activity index in Crohn’s disease, suggesting a more prominent role in repair rather than fuelling inflammatory cascades in this immunologically driven disease. The precise mechanisms to explain the significantly increased plasma levels of the protein during remission compared to active disease remain unclear. Disease activity scores in IBD are based on a combination of clinical and laboratory parameters, which do not incorporate information on mucosal histology^25,34,40^. In a previously symptomatic patient with active disease, resolution of symptoms reflects the onset of clinical remission or inactive disease, which often precedes endoscopic and histological remission^25^. Therefore, the higher plasma levels of periostin during clinical remission may reflect ongoing tissue repair and remodelling which continue even after clinical symptoms have resolved^25,41^. Another explanation could be the upregulation of periostin by transforming growth factor beta (TGF-β), which plays a key role during mucosal healing^2,42^.

Plasma periostin levels differed significantly between the paediatric and adult control participants, despite a difference of only 10.7 years between the median age for the two groups. This is in keeping with previously published literature, demonstrating higher levels in childhood consistent with the activity of bone metabolism^26,30,31^. It has been shown previously that periostin levels show differing trends within the paediatric age group, with a sharp decrease from infancy until five years of age and a mild increase from seven years onwards until age fifteen. In our study, regression analysis did not show a significant association between periostin levels and age among the paediatric patients.

Our observations on immunofluorescence showing enhanced localisation of periostin around the crypts are in keeping with previously described findings in patients with UC^7,8^. Our study presents immunofluorescence findings in both UC and CD, with a similar peri-cryptal distribution pattern in both conditions. Furthermore, persistence of the enhanced peri-cryptal ring pattern was observed in the macroscopically unaffected but histologically inflamed colonic mucosa in CD patients. As a hypothesis, we propose that periostin expression in the GI mucosa could potentially complement routine diagnostic histology as a proxy marker of inflammation. However, further studies will be required to explore this concept in a clinical setting.

Different isoforms were shown to be expressed in different cell types and different circumstances. In our study, no statistically significant difference in the type and the abundance of the different isoforms was observed between UC, CD and healthy controls. However, the possibility that a specific isoform may be responsible for the enhanced peri-cryptal ring pattern observed in paediatric IBD patients cannot be excluded. Also, periostin in these peri-cryptal ring structures may be more stable, what could explain the immuno-histochemistry and PCR findings that there is no dramatic shift in the expressed variants and overall expression levels in inflamed bowel tissue. We also cannot exclude that changes in the expression levels of periostin-binding partners may be responsible for the specific shift in localisation that we observed. To address this specific question, further studies using isoform specific antibodies will be required. If a specific isoform of periostin is responsible for the characteristic signal observed in IBD patients, the specific isoform could potentially be a target for the development of new treatment options.

A major drawback of our study was that the majority of patients were recruited after an established diagnosis of IBD and plasma samples collected at a random time point during the course of the disease. Validated disease activity scores were used to group patients into those with active disease and remission. Given the variability trends observed within the different paediatric age groups as per published literature^43^, an ideal approach would be to compare paired plasma specimens per patient both during active disease and in remission. However, with the unpredictable nature of IBD and given that some patients can remain in prolonged remission (for years), this approach can be challenging. For future research, we propose collecting plasma specimens at diagnosis and at various time points of the disease course in order to assess the differences during active disease and remission.

In this study, the proportion of patients with active CD was approximately 70% compared to 50% of patients with active UC. Whilst it is possible that we did not detect a statistical significance between active disease and remission in the UC cohort due to the smaller sample size, we cannot rule out the possibility of modest effect sizes that could only be detected using a larger sample. Given the lack of significant differences in plasma periostin between patients and age-matched controls, the clinical utility of periostin as a biomarker of inflammation in IBD could not be justified. To this end, the limited size of our paediatric control cohort was clearly a disadvantage. Furthermore, although the control group did not have evidence of ongoing GI pathology, these individuals were originally referred with some GI symptoms. Therefore, strictly speaking, they may not be representative of ‘true healthy controls’.

Despite the similarities in the periostin expression patterns in both CD and UC, a statistically significant difference in plasma periostin levels between the active disease state and remission was only observed in the CD cohort. Although the peri-cryptal ring patterns observed in the two conditions were very similar, our overall impression was that the intensity of the rings was more pronounced in CD compared to UC patients. As we do not have enough immunofluorescence data on patients in remission, we are unclear if explanations for the disparity in plasma levels could be derived if active disease GI specimens were analysed alongside samples in remission. This could potentially be the subject of future research. Also, although CD and UC represent the two distinct phenotypes of IBD, an indistinguishable histological overlap is often present despite the genetic, immunological and phenotypic heterogeneity in the two conditions^44^.

**Figure 6:**
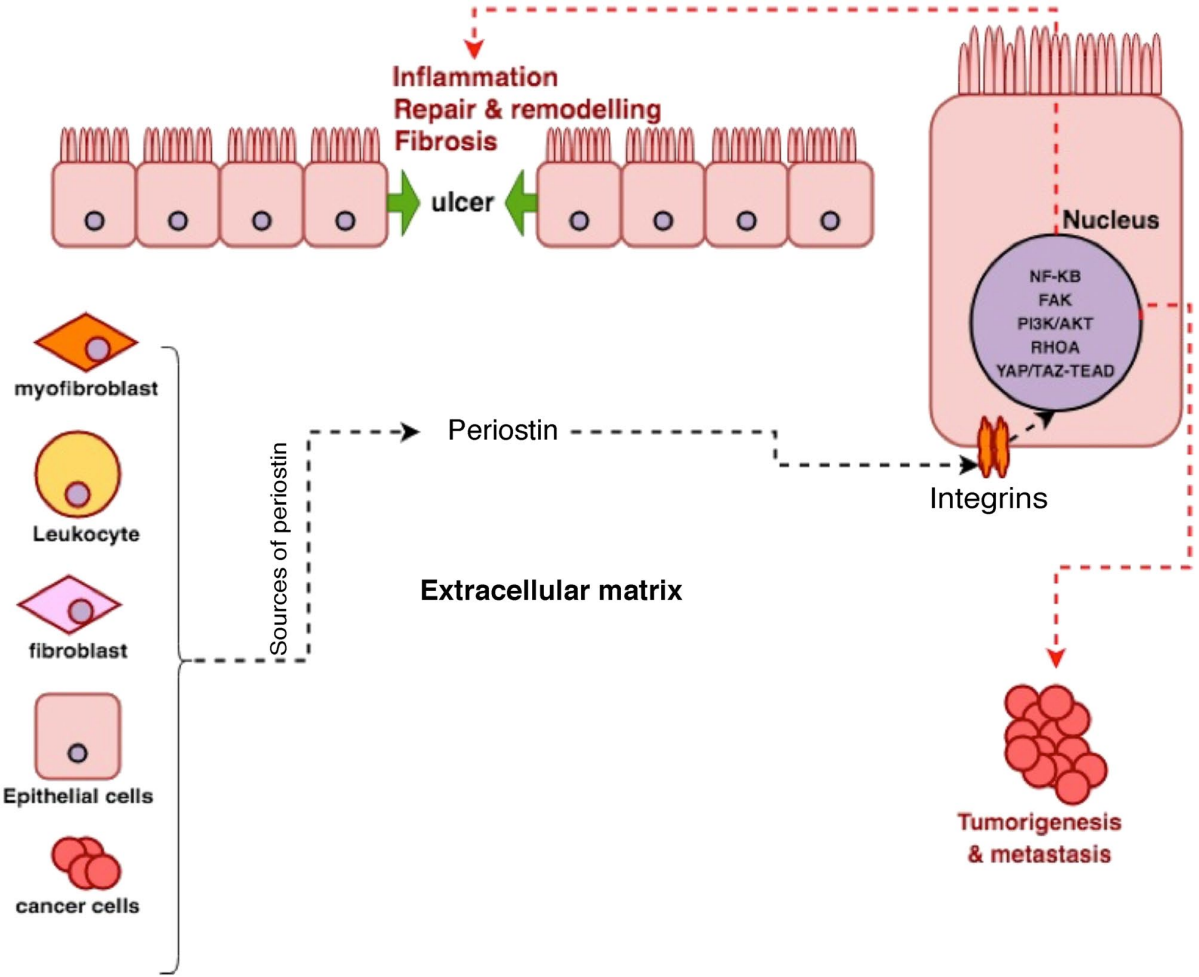
Pathophysiology of periostin. In the tissue, periostin is primarily produced by the fibroblasts and myofibroblasts. It has also been detected in tumour cells, epithelial cells and leukocytes. After engaging with the αv-integrin receptors within the cell membrane, periostin facilitates transmission of signals for cellular adhesion and activation of various immune-signalling pathways. These include the focal adhesion kinase (FAK) phosphatidylinositol 3-kinase (PI3K)/AKT, NF-κB, and RHOA and YAP/TAZ TEAD signalling pathways. These pathways further fuel inflammation and also contribute to tissue repair and remodelling as well as fibrosis and tumorigenesis. This figure was generated by TC using the software https://app.diagrams.net. (Abbreviations: TAZ, transcriptional co-activator with PDZ-binding motif; TEAD, TEA domain family member 1; YAP, Yes-associated protein1).

Periostin can influence the functioning of the NF-κB and several other immune signalling pathways^4,6,7,39^. Through its interaction with the αv-integrin receptors located across the cell membrane, periostin promotes cellular adhesions to the ECM, activates signal transduction and also regulates the expression of several other functionally connected proteins (See Fig. 6). The precise molecular pathways and the functional impact of genomic variation in the periostin network of genes remains elusive. Our paper represents a baseline study investigating the genomic concepts and the immunological profile of periostin during inflammation and repair in IBD. The function of periostin as a matricellular protein, its’ interaction with αv-integrin receptors and the multiple immune signalling pathways makes it a potential target for future treatments in IBD^2,7,25^. Additional studies will be necessary to provide further mechanistic insights and a better understanding of its role in IBD.

## Supplementary Information

Supplementary Information 1.

Supplementary Information 2.
