# Supplementary material for: Expression profile of the matricellular protein periostin in paediatric inflammatory bowel disease
Source: Sci Rep. 2021 Mar 18;11:6194. doi: 10.1038/s41598-021-85096-7 (PMC7973505; doi:10.1038/s41598-021-85096-7)
Supplement: Supplementary file 1 — Supplementary Information 1. [file 41598_2021_85096_MOESM1_ESM.pdf]

Expression Profile of the Matricellular Protein Periostin in Paediatric Inflammatory Bowel Disease

Authors:

$Tracy Coelho (PhD)[1, 3]

$Eva Sonnenberg-Riethmacher (PhD)[2, 4]

Yifang Gao (PhD)[5, 10]

Enrico Mossotto (PhD)[1]

Alisher Khojanazarov[2]

Annie Griffin (PhD)[5]

Saida Mukanova (MD)[2]

Aiymkul Ashimkhanova (MD)[2]

Rachel Haggarty[6]

Anton Borissenko[2]

James J. Ashton (MRCPCH)[1, 3]

Imogen S. Stafford[1]

Akshay Batra (MD)[3]

Nadeem A. Afzal (MD)[3]

Michael P. Stanton (MD)[7]

Bhumita Vadgama (FRCPath)[8]

Kapura Adrisova (MD)[9]

Robert M. Beattie (FRCPCH)[3]

Anthony P. Williams (PhD)[5]

*Sarah Ennis (PhD)[1]

*Dieter Riethmacher (PhD)[2, 4]

$Joint first authors; *Joint corresponding authors

Name of Institutions:

[1] Human Genetics and Genomic medicine, University of Southampton, Southampton, UK

[2] Department of Biomedical Sciences, Nazarbayev University School of Medicine, Nur-Sultan, Kazakhstan

[3] Department of Paediatric Gastroenterology, University Hospital Southampton, UK

[4] Academic Unit of Human Development and Health, University of Southampton, UK

[5] Cancer Sciences Division, Faculty of Medicine, University of Southampton, UK

[6] NIHR, Southampton Biomedical Research Centre, University Hospital Southampton, UK

[7] Department of Paediatric Surgery, University Hospital Southampton, UK

[8] Department of Pathology, University Hospital Southampton, UK

[9] Nazarbayev University Medical Center UMC, Department National Research Center for Maternal and Child Health, Kazakhstan

[10] Organ Transplantation Center, The First Affiliated Hospital, Sun Yatsen University, Guangzhou, China

Correspondence to:

[*]Dr. Dieter Riethmacher

Department of Biomedical Sciences,

Nazarbayev University School of Medicine, Nur-Sultan, Kazakhstan

Telephone: +77172694635 **E-Mail:** dieter.riethmacher@nu.edu.kz

Key words: periostin, inflammatory bowel disease, inflammation

Methods: Supplementary information

Section1. Plasma periostin analysis

Whole blood was collected into commercially available heparinised tubes, transported and stored at room temperature for up to four hours pending plasma extraction. Plasma was separated after centrifugation of whole blood for 10 minutes at 2000 rpm and aliquoted into 0.5ml aliquots for storage at -80 ^{o} C until analysis. Up to 0.5ml of plasma per patient was utilised for periostin analysis. Periostin concentrations in plasma were assessed in duplicate using sandwich ELISA technique (R & D DuoSet human periostin ELISA kit, R & D systems, Abingdon, UK). Plasma periostin analysis was performed using a 96-well microplate, coated with 100μl per well of the diluted capture antibody and incubated overnight at room temperature. Plates were washed three times with the wash buffer provided followed by incubation with reagent diluent for one hour. After a further wash cycle, plates were incubated with samples and standards for two hours at room temperature followed by incubation with detection antibody for 2 hours. The plates were next incubated for 20 minutes with streptavidin-HRP, followed by addition of substrate solution for a further 20 minutes. Colour development was stopped by the addition of 2N H_2SO_4 and the plates were read for optical density using a microplate reader set to 450nm. Sample readings were interpolated on standard curves using GraphPad Prism (V7) programmes, generating a logistic curve-fit and transforming values to concentration units (pg/ml).

Section 2. Immuno-fluorescence staining procedure for periostin

Immuno-fluorescence staining was essentially done as described in Sonnenberg-Riethmacher et al., 2015 [1]. Mucosal biopsies collected during endoscopy were immediately embedded in Optimal Cutting Temperature (OCT) embedding matrix (agar scientific, Essex, UK) in a labelled cryomold and transported in a covered foam cooler of dry ice for storage at -80 ^{o} C

pending analysis. Anti-human periostin (abcam ab14041) were used as the primary antibodies and cy3-labeled anti-rabbit antibody (abcam ab6939) as the secondary antibodies. The frozen block was removed from the -80 ^{o} C freezer, allowed to equilibrate in the cryostat chamber at -20 ^{o} C for approximately 30 minutes. Cryostat sectioning of the tissue (6-8 microns) was performed using anti-roll plate method. Slides were drop fixed in 4% paraformaldehyde (PFA) and phosphate buffer solution (PBS) for 5 minutes followed by a wash cycle in PBS. Fixatives preserve tissue architecture, inactivate proteolytic enzymes that could otherwise degrade the sample, stabilise specimens so that they can withstand further processing and protect samples against microbial contamination [2]. Slides were thereafter treated with PBT (PBS with 0.1% triton) for 10 minutes to allow intracellular staining. To block non-specific binding reactions, sections were incubated with foetal calf serum (50% FCS) in PBT for 1 hour at room temperature. This was followed by overnight incubation of the slides at 4 ^{o} C with the primary antibody (anti-human periostin) diluted in PBT with 10% FCS (1:500 dilution). After wash cycles with PBS, the slides were treated with secondary antibodies for 2 hours at room temperature and washed again 3 times for 5 minutes with PBS. To the second wash DAPI was added for staining of nuclei. 5to 10 sections were analysed per sample. Analysis was done with Axiovert microscope.

Section 3. Periostin isoform analysis

RNA was isolated from intestinal biopsies of patients with ulcerative colitis and crohn's disease as well as non-IBD controls using Qiagen RNeasy kit. RNA was then reverse transcribed into cDNA, which was then used for PCR with one primer specific for Exon 16 (forward primer: CCTTCAAAGAAATCCCCGTGACTGTC) and one reverse primer specific for Exon 23 (reverse primer: TCACTGAGAACGACCTTCCCTTAATC). These primers detect all isoforms of periostin present in a given tissue, as they amplify the entire alternatively spliced area. The PCR mix was analysed by gel electrophoresis (agarose gels

and agilent) and additionally cloned into plasmid pJET1.2/blunt using CloneJet PCR cloning kit (Thermofisher). Subsequently, plasmid DNAs were isolated and sequenced.

Section 4. DNA extraction

Genomic DNA was extracted from peripheral venous blood or saliva specimens using the salting out method [3]. For venous blood specimens, buffy coats of nucleated cells were subjected to cell and nuclear membrane lysis using nuclei lysis buffer (10 mM Tris-HCl, 400 mM NaCl and 2 Mm Na_2EDTA, pH 8.2), cell lysates digested overnight at 37∘ C with 0.2 ml of 10% SDS and 0.5 ml of a protease K solution (1 mg protease K in 1% SDS and 2mM Na_2EDTA), followed by addition of saturated sodium chloride and centrifugation at 2500 rpm for 15 minutes. The supernatant containing the DNA is then treated with absolute ethanol until DNA is precipitated, followed by transfer to micro-centrifuge tube containing 100-200µl TE buffer (10:10 mM Tris-HCl, 400 mM NaCl and 2 Mm Na_2EDTA, pH 7.5). The DNA is allowed to dissolve for 2 hours at 37∘ C, then quantified using the Qubit ® 2.0 Fluorometer and a 260:280 ratio calculated using a nanodrop spectrophotometer. The average DNA yield obtained is 150µg/ml and approximately 20ug of DNA is used for next generation sequencing for each patient.

Section 5. Processing of whole exome sequencing (WES) data

Genomic DNA was extracted from peripheral venous blood (see section 4) and fragmented DNA subjected adaptor ligation and exome library enrichment using the Agilent SureSelect All Exon capture kit versions 4, 5 and 6. Enriched libraries were sequenced on Illumina HiSeq systems. Alignment against the human genome (hg38) was performed using Burrows-Wheeler Aligner (BWA) [4], variants called using Genome Analysis Toolkit (GATK v3.8) and ANNOVAR for variant annotation [5].

Section 6. WES data analysis using GenePy score

WES data analysis was conducted using a whole gene-based pathogenicity score, 'GenePy' as previously described [6]. GenePy incorporates known deleteriousness metrics, allele frequency and individual zygosity information for each variant and sums them to generate a whole gene-based pathogenicity score. Each gene score was subsequently corrected for gene length. In this study, GenePy scores were generated for a list of genes selected for their functional relevance to periostin. For the selection of genes, an electronic search was conducted through 'PathCards', an online database of human biological pathways [7]. PathCards generates a unified set of genes for the interrogated pathway or protein in the form of 'SuperPaths', consolidating biological information from multiple manually curated sources. In this study, the 'SuperPath' nucleating from the search by entering the term 'periostin' was used to select the genes for application of GenePy scores. Interrogation through PathCards using the search term 'periostin' displayed two SuperPaths, 'Amplification and Expansion of Oncogenic Pathways as Metastatic Traits' and 'Hypothesized Pathways in Pathogenesis of Cardiovascular Disease', each comprising seventeen and twenty-five genes respectively, including periostin. The former was selected based on its functional relevance to inflammatory pathologies, given the well-characterised roles of periostin in inflammatory and oncological diseases.

GenePy scores were available for twenty four out of the twenty-seven genes selected. GenePy scores were not available for *CCL5*, *ITGA5B1* and *ITGA5B3* genes. Scores may not be available for all genes as some of the genes may not be properly captured during the exome library capture process, or may not be annotated by the currently available computational databases used when generating GenePy scores.

References:

1. Sonnenberg-Riethmacher E, Miehe M, Riethmacher D. Promotion of periostin expression contributes to the migration of Schwann cells. J Cell Sci 2015;128:3345-3355. doi:10.1242/jcs.174177

2. Shi SR, Liu C, Pootrakul L, et al. Evaluation of the value of frozen tissue section used as "gold standard" for immunohistochemistry. *American journal of clinical pathology* 2008;129(3):358-66. doi: 10.1309/7CXUYXT23E5AL8KQ

3. Miller SA, Dykes DD, Polesky HF. A simple salting out procedure for extracting DNA from human nucleated cells. *Nucleic Acids Res* 1988;16(3):1215.

4. Li H, Durbin R. Fast and accurate short read alignment with Burrows-Wheeler transform. *Bioinformatics* 2009;25(14):1754-60. doi: 10.1093/bioinformatics/btp324

5. Wang K, Li M, Hakonarson H. ANNOVAR: functional annotation of genetic variants from high-throughput sequencing data. *Nucleic acids research* 2010;38(16):e164. doi: 10.1093/nar/gkq603

6. Mossotto E, Ashton JJ, O'Gorman L, et al. GenePy - a score for estimating gene pathogenicity in individuals using next-generation sequencing data. *BMC Bioinformatics* 2019;20(1):254. doi: 10.1186/s12859-019-2877-3

7. Belinky F, Nativ N, Stelzer G, et al. PathCards: multi-source consolidation of human biological pathways. *Database (Oxford)* 2015;2015 doi: 10.1093/database/bav006

Figure S1. Relationship between age and periostin levels

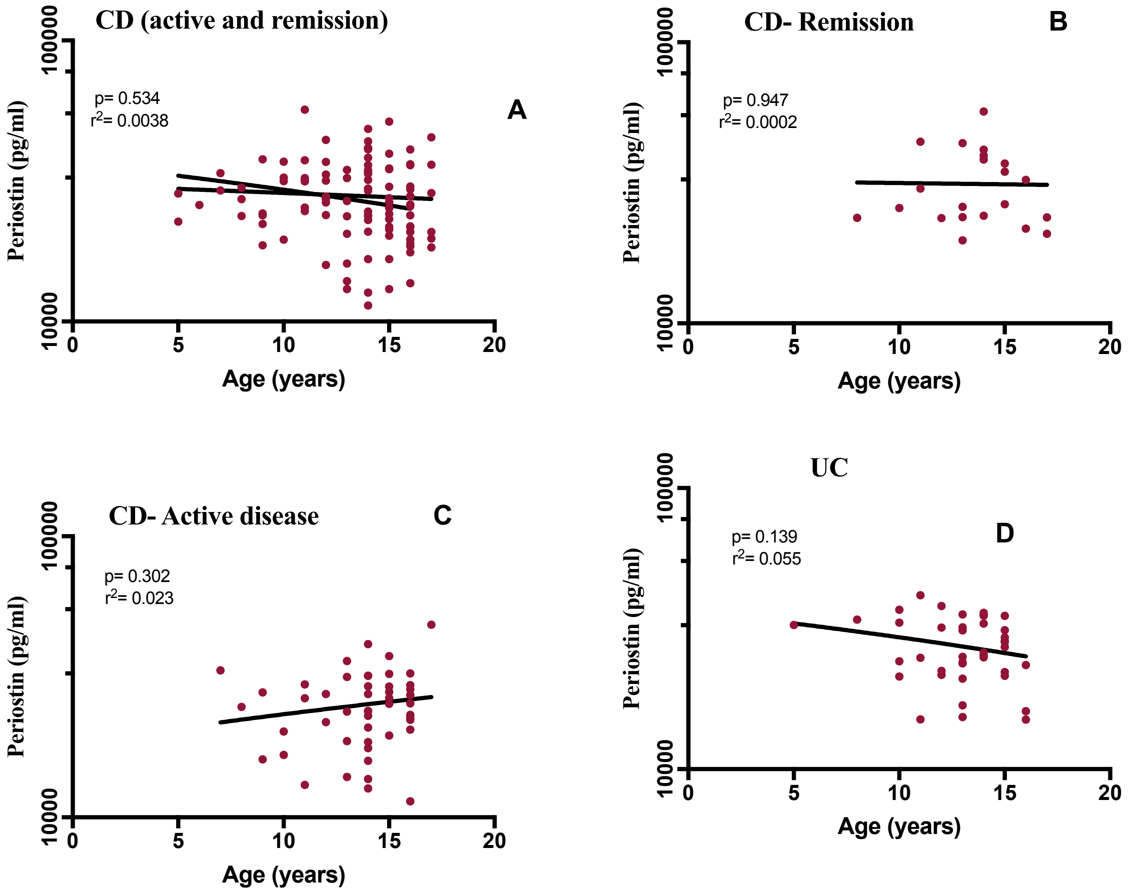

Figure S1. Relationship between age and periostin levels legend:

Age in years is plotted along the X-axis and periostin levels on the Y-axis. A) Patients with Crohn's disease- includes both active disease and those in remission, B) Crohn's disease- patients in remission, C) Crohn's disease- patients with active disease and D) Ulcerative colitis (both active disease & remission). No significant association was observed on regression analysis.

Figure S2. Plasma periostin in the surgical and non-surgical patients

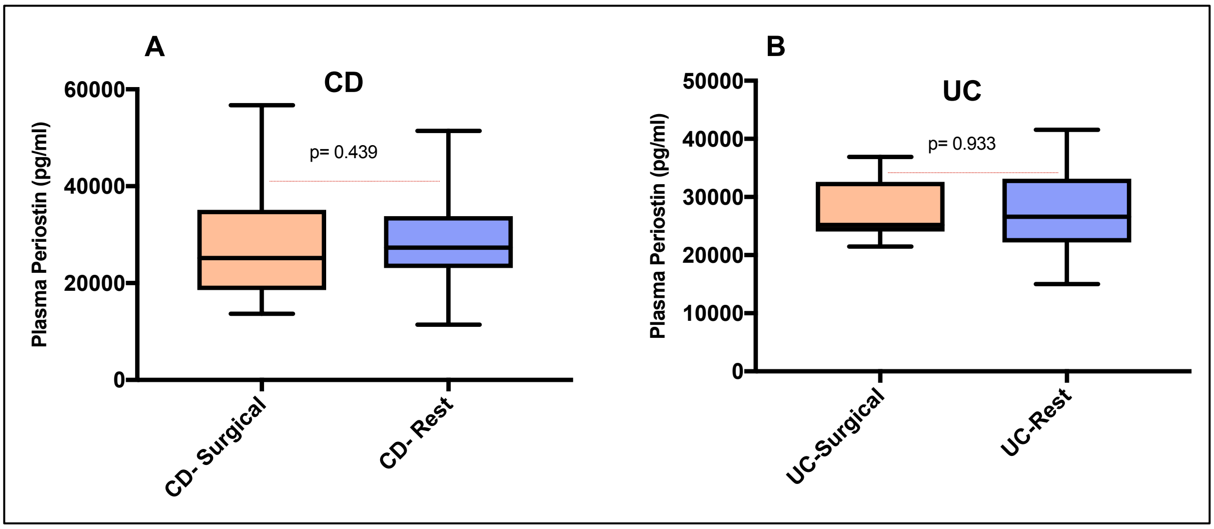

Figure S2. Plasma periostin in the surgical and non-surgical patients legend:

No differences were observed between the surgical and non-surgical patients in both Crohn's disease and ulcerative colitis. A) Crohn's disease (CD) - Plasma periostin in patients who underwent surgery and the rest who did not have surgery B) Ulcerative colitis (UC) - Plasma periostin levels in the surgical and non-surgical groups.

Figure S3. Sequence analysis using GenePy scores

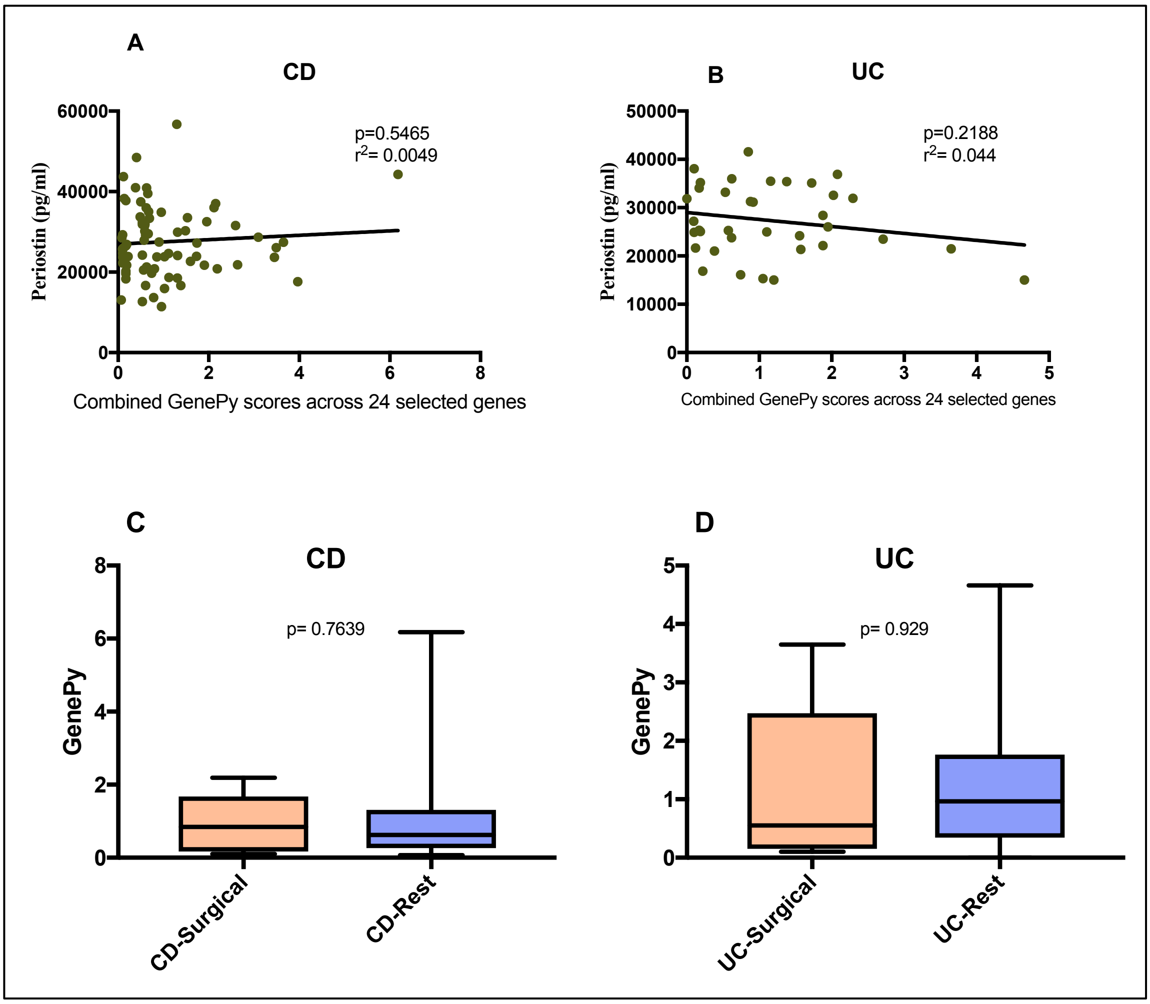

Figure S3. Sequence analysis using GenePy scores legend:

A) Crohn's disease (CD) - No association between plasma periostin levels and combined GenePy scores across 24 selected genes B) Ulcerative colitis (UC) - No association between plasma periostin levels and combined GenePy scores C) CD - No significant differences in GenePy scores in the surgical and non-surgical groups and D) UC - No significant differences in GenePy scores in the surgical and non-surgical groups.

Table S1A. PUCAI Score for ulcerative colitis

PUCAI Score		
Parameter		score
**Abdominal pain**	No Pain	0
	Pain can be ignored	5
	Pain cannot be ignored	10
**Rectal bleeding**	None	0
	Small amounts, in <50% of stools	10
	Small amounts, in most stools	20
	Large amount (>50% of stool content)	30
**Stool consistency of most stools**	Formed	0
	Partially formed	5
	Completely unformed	10
**Number of stools per 24 hours**	0-2 stools	0
	3-5 stools	5
	6-8 stools	10
	>8	15
**Nocturnal stools**	No	0
	Yes	10
**Activity level**	No limitation of activity	0
	Occasional limitation of activity	5
	Severe restricted activity	10
**Total Maximum score**		85

The table shows the scoring parameters for the PUCAI score. The score ranges between 0-85

Table S1B. PCDAI Score for Crohn's disease

	PCDAI Score			
	Parameters	0	5	10
Clinical symptoms and findings	Abdominal pain	None	Mild= brief episodes, not interfering with activities	Moderate/severe, persistent
	Stools	0-1 liquid stools per day, no blood	2-5 liquid stools, small amount of blood	Gross bleeding, > 6 liquid stools or nocturnal diarrhoea
	General Functioning	No limitation of activity	Occasional difficulties in maintaining age-appropriate activities	Frequent limitation of activities
	Weight	Stable weight	involuntary weight loss 1-9%	weight loss >1%
	Height	< 1 channel decrease	1<2 channel decrease	>2 channel decrease
	Abdomen	No tenderness	tenderness, or mass without tenderness	tenderness, involuntary guarding, definite mass
	Peri-rectal disease	none, asymptomatic tags	1-2 indolent fistula, scant drainage	active fistula, drainage, abscess
	Extra-intestinal symptoms, fever >38.5 for 3 days in a week, arthritis, erythema nodosum	None	one	two
	Albumin	>35	31-34	<30
	Score			
		0	2.5	5
Haematocrit	< 10 years	>33	28-33	<28
	11-14 (male)	>35	30-34	<30
	15-19 (male)	>37	32-36	<32
	11-19 (female)	>34	29-33	<29
	ESR	<20	20-50	>50

The table shows the PCDAI score. The score ranges between 0-100.

Table S2A-Paris Classification- CD

ID	Age	Location	Behaviour	PSC IBD	Growth	PCDAI	Disease
CD1	1b	L3	B1B4	No	N/A	20	Active
CD98	1b	N/A	N/A	No	N/A	5	Remission
CD3	1b	L3	B1	No	N/A	25	Active
CD43	1b	L3L4	N/A	No	N/A	5	Remission
CD51	1a	L3	B1	No	N/A	Unknown	Unknown
CD92	1b	L3L4	B1	No	N/A	40	Active
CD93	1b	L3L4	B1	No	N/A	20	Active
CD8	1a	L3L4	B1	No	N/A	30	Active
CD97	1a	L3	N/A	No	N/A	5	Remission
CD10	1a	L3	N/A	No	N/A	35	Active
CD11	1a	L1	N/A	No	N/A	5	Remission
CD12	1b	N/A	N/A	Yes	N/A	10	Active
CD13	1b	L2	N/A	No	N/A	5	Remission
CD14	1b	L1	B2B3	No	N/A	5	Remission
CD15	1a	L3	B2	No	N/A	Unknown	Unknown
CD16	1b	L1	B2	No	N/A	5	Remission
CD17	1b	L1	N/A	No	N/A	Unknown	Unknown
CD18	1a	L2	N/A	No	N/A	25	Active
CD19	1b	L3	B2	No	N/A	Unknown	Unknown
CD20	1b	N/A	N/A	No	N/A	Unknown	Unknown
CD21	1b	L3	B2B3	No	N/A	10	Active
CD22	1b	L1	B2B3	No	N/A	Unknown	Unknown
CD23	1b	L3L4	B3B4	No	N/A	25	Active
CD24	1b	L2L4	N/A	No	N/A	20	Active
CD25	1a	L3	B1B4	No	N/A	5	Remission
CD26	1a	L2	B3B4	No	N/A	Unknown	Unknown
CD27	1a	L3L4	N/A	No	N/A	Unknown	Unknown
CD28	1b	N/A	N/A	No	N/A	Unknown	Unknown
CD29	1b	L1L4	N/A	No	N/A	Unknown	Unknown
CD30	1b	N/A	N/A	No	N/A	20	Active
CD31	1b	L1	N/A	No	N/A	Unknown	Unknown
CD32	1a	L1	N/A	No	N/A	Unknown	Unknown
CD33	1b	L1	N/A	No	N/A	35	Active
CD34	1b	N/A	N/A	No	N/A	10	Active
CD35	1b	L1L4	N/A	No	N/A	Unknown	Unknown
CD36	1b	L2L4	B1	No	N/A	10	Active
CD37	1b	L3L4	N/A	No	N/A	20	Active
CD38	1b	L1	N/A	No	N/A	5	Remission
CD39	1b	L3L4	N/A	No	N/A	Unknown	Unknown
CD40	1b	L1	N/A	No	N/A	10	Active
CD41	1b	L3L4	N/A	No	N/A	Unknown	Unknown
CD42	1b	N/A	N/A	No	N/A	5	Remission
CD4	1b	L3	B3	No	N/A	25	Active
CD44	1b	L3	N/A	No	N/A	Unknown	Unknown
CD45	1a	L2	N/A	No	N/A	0	Remission
CD46	1b	L3	N/A	No	N/A	20	Active
CD47	1b	L1	N/A	No	N/A	Unknown	Unknown
CD48	1b	L3	B3	No	N/A	20	Active
CD49	1b	L2	N/A	No	N/A	5	Remission
CD50	1b	L1	N/A	No	N/A	Unknown	Unknown
CD5	N/A	N/A	N/A	No	N/A	20	Active
CD52	1a	L2L4	B3B4	No	N/A	5	remission
CD53	1a	L2	B1B4	No	N/A	5	Remission
CD54	1b	L2	B1	No	N/A	5	Remission
CD55	1b	L1	B1	No	N/A	20	Active
CD56	1b	N/A	N/A	No	N/A	5	Remission
CD57	1b	L2	N/A	No	N/A	25	Active
CD58	1b	L2L4	B1	No	N/A	15	Active
CD59	1a	L3L4	B1	No	N/A	Unknown	Unknown
CD60	1a	L3	B1	No	N/A	5	Remission
CD61	1b	L2L4	B1	No	N/A	5	Remission
CD62	1b	L4a	B1	No	N/A	Unknown	Unknown
CD63	1b	L3	B1	No	N/A	Unknown	Unknown
CD64	1a	L1	B2B4	No	N/A	15	Active
CD65	1b	L2L4	B3	No	N/A	35	Active
CD66	1b	L2L4	B1	No	N/A	25	Active
CD67	1b	L1L4	B1	No	N/A	Unknown	Unknown
CD68	1b	L3	B2	No	N/A	30	Active
CD69	1b	L3L4	N/A	No	N/A	Unknown	Unknown
CD70	1b	L1	B1	No	N/A	25	Active
CD71	1b	L3L4	B1	No	N/A	30	Active
CD72	1b	L3	N/A	No	N/A	Unknown	Unknown
CD73	1b	L3L4	B1	No	N/A	10	Active
CD74	1a	L3L4a	B1	No	N/A	25	Active
CD75	1b	L3	B1B4	No	N/A	Unknown	Unknown
CD76	1b	L3L4	B1	No	N/A	10	Active
CD77	1b	L3	B1	No	N/A	5	Remission
CD78	1a	L3L4	B1B4	No	N/A	10	Active
CD79	1b	L3L4	B1	No	N/A	Unknown	Unknown
CD80	1b	N/A	N/A	No	N/A	Unknown	Unknown
CD81	1b	L2	B1	No	N/A	45	Active
CD82	1b	L3L4	B1	No	N/A	20	Active
CD83	1b	L3	B1	No	N/A	Unknown	Unknown
CD84	1a	L3	B1	No	N/A	15	Active
CD85	1b	L2	B1	No	N/A	5	Remission
CD86	1b	L3L4a	B1B4	No	N/A	50	Active
CD87	1b	N/A	N/A	No	N/A	20	Active
CD88	1a	N/A	N/A	No	N/A	Unknown	Unknown
CD89	1b	L3	B1	No	G0	Unknown	Unknown
CD90	1b	L2L4a	B1	No	G0	35	Active
CD91	1b	L3	B1	No	G0	50	Active
CD6	1b	L3	B3	No	G1	35	Active
CD7	1b	L3L4a	B1	No	N/A	35	Active
CD94	1b	L2	B1	No	G0	40	Active
CD95	1b	L3	B1	No	G0	Unknown	Unknown
CD96	1a	L2L4a	B1	No	G0	Unknown	Unknown
CD9	1a	L2	B1	No	G0	35	Active
CD2	1b	L4a	B1	No	G0	30	Active
CD99	1b	L3L4a	B1	No	G0	25	Active
CD100	1b	L3	B1B4	No	G0	45	Active
CD101	1b	N/A	N/A	No	N/A	5	Remission
CD102	1b	L3	B1	No	G0	Unknown	Unknown

Age at Diagnosis (yrs)	1a = 0 to less than 10; 1b = 10 to less than 17;
Location	Number only: L1 = terminal ileum, L2= colon, L3 = ileocolon, L4a = upper GI proximal to Ligament of Treitz, L4b= upper GI distal to Ligament of Treitz and proximal to distal 1/3 ileum, L1L4 = terminal ileum, L2L4a = colon & upper GI, L3L4a = ileocolon & upper GI, L1L2=terminal ileum and colon
Behaviour	Number only: 1 = nonstricturing, nonpenetrating, 2= stricturing, 3 = penetrating, 14 = nonstricturing, nonpenetrating & perianal, 24= stricturing & perianal, 34 = penetrating, perianal 23=stricturing and penetrating, p= perianal
Growth	0= No evidence of growth delay,1 = Growth delay
PSC IBD	Primary sclerosing cholangitis

Table S2B - Paris Classification - UC

ID	Age	Extent	PSC	PUCAI	Disease state
UC5	1a	E4	No	0	Remision
UC1	1b	E4	No	75	Active
UC3	1b	E4	No	0	Remision
UC4	1a	E4	No	0	Remision
UC2	1b	E4	No	70	Active
UC6	1a	E4	No	25	Active
UC7	1a	N/A	No	45	Active
UC8	1a	N/A	No	45	Active
UC9	1b	N/A	No	60	Active
UC10	1b	E4	No	70	Active
UC11	1a	E4	No	45	Active
UC12	1b	N/A	No	5	Remision
UC13	1b	E2	No	5	Remision
UC14	1b	N/A	No	0	Remision
UC15	1b	E4	No	10	Active
UC16	1a	E1	No	15	Active
UC17	1b	E2	No	20	Active
UC18	1b	N/A	No	0	Remision
UC19	1b	N/A	No	0	Remision
UC20	1a	E4	No	0	Remision
UC21	1b	E4	No	5	Remision
UC22	1b	E2	No	25	Active
UC23	1b	N/A	No	0	Remision
UC24	1b	E4	No	10	Active
UC25	1b	E2	No	5	Remision
UC26	1b	E1	No	5	Remision
UC27	1a	E4	No	5	Remision
UC28	1b	E4	No	25	Active
UC29	1b	N/A	No	20	Active
UC30	1b	E4	No	0	Remision
UC31	1b	E4	No	40	Active
UC32	1b	E1	No	70	Active
UC33	1b	N/A	No	20	Active
UC34	1b	E2	No	15	Active
UC35	1b	E4	No	0	Remision
UC36	1b	E4	No	0	Remision
UC37	1b	E4	No	0	Remision
UC38	1b	E4	No	0	Remision
UC39	1b	E1	No	0	Remision
UC40	1b	E2	No	65	Active
UC41	1b	E4	No	5	Remision
UC42	1b	E3	No	20	Active

Age at Diagnosis (yrs)	1a = 0 to less than 10; 1b = 10 to less than 17;
Extent	Number only: 1 = ulcerative proctits, 2 = left-sided, 3 = extensive, 4=Pancolitis

[illegible]

ID	Gender	Periostin pg/ml	Surgery
Crohn's disease			
CD-26	F	27480	Terminal ileal resection + ileostomy, adhesiolysis
CD-1	M	41497	Right hemicolectomy
CD-23	M	56725	sigmoid Strictures, dilatation
CD-60	M	37795	Terminal ileal strictures, ileostomy
CD61	M	36009	Mid- transverse Colostomy, rectal stricture, ileostomy
CD62	F	21748	De-functioning ileostomy
CD63	M	20829	Right hemicolectomy
CD64	F	30252	Ileo-caecal resection for stictures with ileostomy
CD65	F	18659	Diversion ileostomy
CD66	F	19609	Right hemicolectomy
CD67	F	26559	Ileo-caecal resection for stictures, right hemi-colectomy
CD68	F	37012	ileostomy
CD69	F	13692	Right hemicolectomy
CD-70	M	32378	Right hemicolectomy
CD-72	M	18340	Colectomy with ileostomy
CD-74	M	36992	Colectomy with ileostomy
CD-58	M	13912	colectomy with ileostomy
Ulcerative colitis			
UC-16	F	31159	Sub-total colectomy + ileostomy
UC-14	F	36908	Sub-total colectomy + ileostomy
UC-15	M	25074	Sub-total colectomy + ileostomy
UC-22	M	25331	Sub-total colectomy + ileostomy
UC-29	F	24901	Sub-total colectomy + ileostomy
UC-34	M	21480	Sub-total colectomy + ileostomy

Table S4 GenePy scores - CD

Sample ID			Sum Scores	*CCR5*	*CXCR4*	*CYTIP*	*EPAS1*	*IL6*	*JAG1*	*LEF1*	*MMP2*	*MMP9*	*NOTCH1*	*PIK3CG*	*POSTN*	*RHOA*	*ROCK1*	*SRC*	*STAT3*	*TCF7*	*TCF7L1*	*TCF7L2*	*TNC*	*VCAM1*	*VEGFA*	*VHL*	*WNT2*
Gene Lengt	Surgery, no=1, yes=2	Periostin (pg/ml)		1044	1247	1681	3202	967	4933	2265	2316	2582	8864	3587	3913	936	5239	2076	3915	2856	2303	3367	7148	2141	2189	872	1200
CD1	1	20840	0.0008802	0	0	0	0.0001393	0	0.000102	0	0.0002621	8.009E-05	6.609E-05	0.0001129	0	0	0	4.104E-10	0	8.017E-05	0	0	3.093E-05	6.641E-06	0	0	0
CD2	1	27441	0.0027176	0	0	0	0.0001186	0.000578	7.29E-05	0.000311	8.342E-05	8.82E-05	0.000237	0.0003646	0	0	0	4.104E-10	0	0	0	0.0002386	0.0004671	0.0001582	0	0	0
CD3	1	23687	0.001771	0	0	0	0	0	0	0	0.0002593	0.0003716	4.986E-05	0.0001636	0	0	0	4.104E-10	0	0.0005309	0	0	0.0002653	6.641E-06	0.0001239	0	0
CD4	1	12662	0.0008974	0	0	0	0	0	0.0001706	0	0.0002621	0.000155	5.496E-05	5.575E-05	0	0	0	4.104E-10	0	9.544E-05	0	0	9.692E-05	6.641E-06	0	0	0
CD5	1	43740	0.0011921	0.0004292	0	0	0	0	0.000103	0	8.342E-05	8.009E-05	4.986E-05	6.154E-06	0	0	0	4.104E-10	0	0	0	0	0.0003099	6.641E-06	0.0001239	0	0
CD6	1	24131	0.0016689	0	0.0002925	0	0	0	0.000227	0	0.0002721	8.009E-05	5.496E-05	6.154E-06	0	0	0.000109	4.104E-10	0	9.544E-05	0.0001931	0.0002003	0.0001316	6.641E-06	0	0	0
CD7	1	37453	0.0005283	0	0	0	0	0	0.0001231	0	8.342E-05	8.009E-05	2.045E-05	0.000114	0	0	0	4.104E-10	0	0	0	0	0.0001007	6.641E-06	0	0	0
CD8	1	40909	0.0018971	0.0004292	0	0	0	0	3.655E-05	0	0.0004385	0.000155	3.854E-05	0.0002107	0	0	0	4.104E-10	0	0	0	0	0.0001663	0.0004224	0	0	0
CD9	1	26852	0.0010494	0	0	0	0	0	0.0002217	0	0.0002378	0.0001768	9.127E-05	6.154E-06	0	0	0	4.104E-10	0	0	0	0	0.0001851	6.641E-06	0.0001239	0	0
CD10	1	13050	0.0011642	0.0004292	0	0	0	0	0.0001184	0	0	0.0003716	0	8.055E-05	0	0	0	4.104E-10	0	0	0	0	0.0001578	6.641E-06	0	0	0
CD11	1	21264	0.0019773	0.0004292	0	0	0.0002599	0	0.0002605	0	0.0002378	0.0005101	1.536E-05	6.154E-06	0	0	0	4.104E-10	0	0	0	0	0.0001277	6.641E-06	0.0001239	0	0
CD12	1	28672	0.0015051	0	0	0	0	0	0.000107	0	0.0002378	0.0001851	0.0001291	0.0002107	0	0	0	4.104E-10	0	0.0001497	0.0001931	0	0.000286	6.641E-06	0	0	0
CD13	1	31598	0.0015625	0	0	0	0.0002599	0	0.0001403	0	0.0002378	1.31E-05	5.496E-05	0.0004604	0	0	0	4.104E-10	0	0	0	0	0.0002654	6.641E-06	0.0001239	0	0
CD14	1	11402	0.0016171	0	0	0	0	0	0.0002929	0	0.0003036	0.000155	0.0001312	0.0003029	0	0	0	4.104E-10	0	0.0003133	0	0	0.0001117	6.641E-06	0	0	0
CD15	1	38295	0.0012479	0.0004292	0	0	0	0	0.0001365	0	0.0002621	8.82E-05	5.496E-05	8.055E-05	0	0	0	4.104E-10	0	0	0	0	0.0001898	6.641E-06	0	0	0
CD16	1	26073	0.0019741	0.0004292	0	0	0	0.0004105	0.0001555	0	0.0002378	8.009E-05	0.0001425	5.575E-05	0	0	0.0003011	4.104E-10	0	0	0	0	0.000155	6.641E-06	0	0	0
CD17	1	29934	0.0015032	0	0	0	0	0	0.0001363	0.0007723	0	0.0001117	4.986E-05	6.154E-06	0	0	0	4.104E-10	0	9.544E-05	0.0001931	0	0.0001316	6.641E-06	0	0	0
CD18	1	26543	0.0007857	0	0	0	0	0	0.000186	0	0	8.009E-05	4.874E-05	0.0001961	0	0	0	4.104E-10	0	0	0	0	0.0001442	6.641E-06	0.0001239	0	0
CD19	1	35992	0.0014193	0	0.0002925	0	0	0	0.0001184	0	0.0002378	0.000155	5.496E-05	0.0002243	0	0	0	4.104E-10	0	0	0	0	0.0002058	6.641E-06	0.0001239	0	0
CD20	1	48493	0.0011859	0	0	0	0	0	0.0002292	0	0.0002378	0.0003716	9.637E-05	0.0001747	0	0	0	4.104E-10	0	0	0	0	6.964E-05	6.641E-06	0	0	0
CD21	1	21748	0.000404	0	0	0	0	0	6.881E-05	0	0	0.000155	3.451E-05	6.154E-06	0	0	0	4.104E-10	0	0	0	0	0.0001328	6.641E-06	0	0	0
CD22	1	31547	0.0019548	0	0	0	0.0001393	0	0.000103	0	0.000934	0.0003048	5.496E-05	0.0001747	0	0	0	4.104E-10	0	0	0	0	0.0001137	6.641E-06	0.0001239	0	0
CD23	1	20202	0.0017377	0.0004292	0	0	0.0001393	0	0	0	0.0002378	0.000155	5.113E-05	6.154E-06	0	0	0	0.0003798	0	0	0	0	0.0003327	6.641E-06	0	0	0
CD24	1	33731	0.0008226	0	0	0	0	0	0	0	0.0002378	0.000155	7.119E-05	0.0002243	0	0	0	4.104E-10	0	0	0	0	0.0001277	6.641E-06	0	0	0
CD25	1	24628	0.0013577	0.0006515	0	0	0	0	6.881E-05	0	0	0.000155	5.113E-05	6.154E-06	0	0	0	4.104E-10	0	0	0	0.0002473	0.0001713	6.641E-06	0	0	0
CD26	1	44275	0.0018996	0	0	0	0	0	0.0003019	0	0	0.000155	2.829E-05	6.154E-06	0	0	0	4.104E-10	0	0	0	0	0.0001007	6.641E-06	0.0001239	0	0.001177
CD27	1	40991	0.0006311	0	0	0	0	0	0.0001572	0	0	0.0001768	4.986E-05	5.575E-05	0	0	0	4.104E-10	0	0	0	0	0.0001848	6.641E-06	0	0	0
CD28	1	31874	0.0009482	0	0	0	0	0	3.655E-05	0	0.0002378	8.009E-05	4.603E-05	0.0001747	0	0	0.0001646	4.104E-10	0	0	0	0	0.0002019	6.641E-06	0	0	0
CD29	1	20524	0.0007858	0	0	0	0	0	0.0001184	0	8.342E-05	0.0001117	7.119E-05	0.0002243	0	0	0	4.104E-10	0	0	0	0	0.0001702	6.641E-06	0	0	0
CD30	1	39544	0.0015364	0.0006515	0	0	0	0	0.0003298	0	0	1.31E-05	7.629E-05	0.0002243	0	0	0	4.104E-10	0	0	0	0	0.0002348	6.641E-06	0	0	0
CD31	1	22663	0.0026311	0.0010422	0	0	0	0	0.000103	0	0.0002621	0.0003198	0.000276	0.0001747	0	0	0	4.104E-10	0	0	0	0	0.0003229	6.641E-06	0.0001239	0	0
CD32	1	30173	0.0013617	0.0004292	0	0	0	0	0.0001363	0	0.0002378	0.0001768	5.496E-05	5.575E-05	9.363E-05	0	0	4.104E-10	0	0	0	0	0.0001706	6.641E-06	0	0	0
CD33	1	23167	0.0010272	0	0	0	0	0	0.000107	0	0.0004878	8.009E-05	0.0001247	6.154E-06	0	0	0	4.104E-10	0	0	0	0	6.313E-05	0.0001582	0	0	0
CD34	1	24692	0.0007955	0	0	0	0	0	0.0001403	0	0.0002378	8.009E-05	7.629E-05	0.0001961	0	0	0	4.104E-10	0	0	0	0	5.821E-05	6.641E-06	0	0	0
CD35	1	34853	0.0006612	0	0	0	0	0	0.0001211	0	0.0002378	8.009E-05	2.045E-05	6.154E-06	0	0	0	4.104E-10	0	8.017E-05	0	0	0.0001088	6.641E-06	0	0	0
CD36	1	19730	0.0009815	0	0	0	0	0	0.0003298	0	0	0.0001117	0.0001247	0.0002107	0	0	0	4.104E-10	0	0	0	0	0.0001979	6.641E-06	0	0	0
CD37	1	25967	0.001199	0	0	0	0	0	0.0002386	0	0.0002378	0.000155	0.0001105	0.0003029	0	0	0	4.104E-10	0	0	0	0	0.0001476	6.641E-06	0	0	0
CD38	1	32535	0.0016529	0	0	0	0	0	0.0002386	0.000311	0.0001887	8.009E-05	5.496E-05	0.0003646	0	0	0	4.104E-10	0	0	0	0	0.0001328	0.0001582	0.0001239	0	0
CD39	1	31874	0.0012322	0	0	0.000615	0	0	0.0001922	0	0	8.009E-05	0	5.575E-05	0	0	0	4.104E-10	0	9.544E-05	0	0	6.313E-05	6.641E-06	0.0001239	0	0
CD40	1	27938	0.0014126	0.0004292	0	0	0	0	0.0001386	0	0.0002378	8.009E-05	7.741E-05	0.0001747	0	0	0	4.104E-10	0	0	0	0	0.0001442	6.641E-06	0.0001239	0	0
CD41	1	33353	0.0005675	0	0	0	0	0	0	0	0.0001887	0.000155	1.809E-05	5.575E-05	0	0	0	4.104E-10	0	8.017E-05	0	0	6.313E-05	6.641E-06	0	0	0
CD42	1	33545	0.0021945	0.0004292	0	0	0	0	0.000211	0	0.0003485	0.0003716	8.251E-05	0.0001747	9.363E-05	0	0	4.104E-10	0	8.017E-05	0	0	0.0001693	6.641E-06	0.0002272	0	0
CD43	1	16663	0.0021436	0	0	0	0	0.0005812	0.0001403	0	0.0002621	0.0002519	5.987E-05	5.575E-05	0.0006889	0	0	4.104E-10	0	0	0	0	9.692E-05	6.641E-06	0	0	0
CD44	1	23841	0.0022801	0	0.0002925	0	0	0	3.655E-05	0	0.0003485	0.0011248	2.045E-05	5.575E-05	0	0	0	4.104E-10	0	0	0.0001931	0	0.0002019	6.641E-06	0	0	0
CD45	1	23784	0.0009578	0	0	0	0	0	0.000324	0	0.0002378	8.009E-05	4.29E-05	5.575E-05	0	0	0	4.104E-10	0	0	0	0	0.0002106	6.641E-06	0	0	0
CD46	1	29257	0.0004648	0	0	0	0	0	0.0001403	0	8.342E-05	8.009E-05	2.045E-05	6.154E-06	0	0	0	4.104E-10	0	0	0	0	0.0001277	6.641E-06	0	0	0
CD47	1	35010	0.0011122	0	0	0	0	0	0.0004462	0	0	0.0003716	5.496E-05	5.575E-05	0	0	0	4.104E-10	0	8.017E-05	0	0	9.692E-05	6.641E-06	0	0	0
CD48	1	24247	0.0013105	0	0	0	0	0	0.0002721	0	0.0003036	0.0004451	0.0001183	6.154E-06	0	0	0	4.104E-10	0	9.544E-05	0	0	6.313E-05	6.641E-06	0	0	0
CD49	1	21829	0.001465	0	0	0	0	0	0.0001184	0	0.0002378	0.000155	9.622E-05	0.0006431	0	0	0	4.104E-10	0	8.017E-05	0	0	0.0001277	6.641E-06	0	0	0
CD50	1	28544	0.0008687	0	0	0	0	0	3.655E-05	0	0.0003772	0.000155	0.0001595	6.154E-06	0	0	0	4.104E-10	0	0	0	0	0.0001277	6.641E-06	0	0	0
CD51	1	15906	0.0010275	0	0	0	0	0	5.34E-05	0	0.0002378	0.000155	0	5.575E-05	0	0	0	4.104E-10	0	0	0.0001931	0	0.0002019	6.641E-06	0.0001239	0	0
CD52	1	23895	0.0013138	0.0004292	0	0	0	0	0.000102	0	0.0003772	8.009E-05	6.099E-05	5.575E-05	0	0	0	4.104E-10	0	0	0	0	0.0002019	6.641E-06	0	0	0
CD53	1	23938	0.0012239	0	0	0	0	0	3.655E-05	0	8.342E-05	0.0003716	4.874E-05	6.154E-06	0.0003916	0	0.000109	4.104E-10	0	0	0	0	0.0001702	6.641E-06	0	0	0
CD54	1	17646	0.0023312	0	0.0002925	0.0009365	0	0	0.0001386	0	0.0003727	0.0002449	4.986E-05	5.575E-05	0	0	0	4.104E-10	0	0	0	0	0.0002337	6.641E-06	0	0	0
CD55	1	29218	0.0018631	0.0006515	0	0	0.0001393	0	0.0002217	0	0.0004385	8.009E-05	1.809E-05	0.0001961	0	0	0	4.104E-10	0	8.017E-05	0	0	3.093E-05	6.641E-06	0	0	0
CD56	1	25717	0.0006534	0	0	0	0	0	0	0	0.0001544	0.000155	0.00015	5.575E-05	0	0	0	4.104E-10	0	0	0	0	0.0001316	6.641E-06	0	0	0
CD57	1	23745	0.0009791	0	0	0	0	0	0.0001816	0	0.0001542	0.0003283	1.536E-05	0.0001961	0	0	0	4.104E-10	0	0	0	0	9.692E-05	6.641E-06	0	0	0
CD58	2	27480	0.001123	0	0	0	0	0	0.0001922	0	0.0001542	8.009E-05	3.068E-05	8.055E-05	0	0	0	4.104E-10	0	0.0002579	0.0001931	0	0.0001277	6.641E-06	0	0	0
CD59	1	29535	0.0015177	0	0	0.000513	0.0002599	0	0.0001747	0	0.0002378	8.009E-05	0.0001376	6.154E-06	0	0	0	4.104E-10	0	0	0	0	0.0001018	6.641E-06	0	0	0
CD60	2	56725	0.0012611	0	0	0	0	0	0.0002646	0	8.342E-05	0.0002603	5.496E-05	0.0003251	0	0	0	4.104E-10	0	9.544E-05	0	0	0.0001706	6.641E-06	0	0	0
CD61	2	37795	0.0014398	0.0006515	0	0	0	0	0.000103	0	0.0002378	8.009E-05	0.0001338	5.575E-05	0	0	0	4.104E-10	0	0	0	0	0.0001713	6.641E-06	0	0	0
CD62	2	36009	0.0010585	0	0	0	0.0003111	0	0.0001403	0	0	0.0003716	1.809E-05	5.575E-05	0	0	0	4.104E-10	0	0	0	0	0.000155	6.641E-06	0	0	0
CD63	2	21748	0.0016089	0.0004292	0	0	0	0	6.881E-05	0	0	0.0003048	3.232E-05	0.0001747	9.363E-05	0	0	4.104E-10	0	0	0	0.0002473	0.0001277	6.641E-06	0.0001239	0	0
CD64	2	20829	0.0012924	0	0	0	0	0	0.0001184	0	0	0.000155	7.629E-05	6.154E-06	0.0003269	0	0	4.104E-10	0	0	0.0004753	0	0.0001277	6.641E-06	0	0	0
CD65	2	30252	0.0007973	0	0	0	0	0	6.881E-05	0	0	8.009E-05	7.642E-05	0.0002243	0	0	0	4.104E-10	0	0	0	0	0.0003411	6.641E-06	0	0	0
CD66	2	18659	0.0010772	0	0	0	0	0	0.0001747	0	0.0002378	1.31E-05	0.0001804	0.0001636	0	0	0	4.104E-10	0	8.017E-05	0	0	9.692E-05	6.641E-06	0.0001239	0	0
CD67	2	19609	0.0011266	0	0	0	0	0	0.0001753	0	0.0003279	0.0003716	1.809E-05	5.575E-05	0	0	0	4.104E-10	0	0	0	0	0.0001713	6.641E-06	0	0	0
CD68	2	26559	0.0007766	0	0	0	0	0	6.881E-05	0	0.0003279	8.009E-05	8.251E-05	5.575E-05	0	0	0	4.104E-10	0	0	0	0	0.000155	6.641E-06	0	0	0
CD69	2	37012	0.0015415	0	0.0002925	0	0	0	7.544E-05	0	0.0001887	8.009E-05	0.0001167	0.0002825	0	0	0	4.104E-10	0	8.017E-05	0	0	0.0004188	6.641E-06	0	0	0
CD70	2	13692	0.0009684	0	0	0	0	0	0.000107	0	0.0002378	0.000155	0.0001058	0.0003251	0	0	0	4.104E-10	0	0	0	0	3.093E-05	6.641E-06	0	0	0
CD71	1	16674	0.0008352	0	0	0	0	0	0.0001363	0	0.0003279	8.009E-05	9.047E-05	5.575E-05	0	0	0	4.104E-10	0	8.017E-05	0	0	5.796E-05	6.641E-06	0	0	0
CD72	2	32378	0.0012296	0	0.0002925	0	0	0	0.0001517	0	0.0003485	0.000155	6.099E-05	5.575E-05	0	0	0	4.104E-10	0	9.544E-05	0	0	6.313E-05	6.641E-06	0	0	0
CD73	1	27243	0.0008707	0	0	0	0	0	9.084E-05	0	0	0.000155	0.0001032	6.154E-06	9.363E-05	0	0	4.104E-10	0	8.017E-05	0	0	0.0002112	6.641E-06	0.0001239	0	0
CD74	2	18340	0.0005642	0	0	0	0.0001393	0	0.000107	0	0	8.009E-05	2.045E-05	5.575E-05	0	0	0	4.104E-10	0	0	0	0	0.000155	6.641E-06	0	0	0
CD75	1	22257	0.0009935	0	0	0	0.0001393	0	0.0001231	0	0.0003485	0.000155	4.986E-05	0.0001129	0	0	0	4.104E-10	0	0	0	0	5.821E-05	6.641E-06	0	0	0
CD76	1	18520	0.0013674	0	0	0	0	0	0.0001403	0	0.0002378	0.0003716	5.496E-05	0.0001747	0	0	0	4.104E-10	0	0	0.0001931	0	0.0001882	6.641E-06	0	0	0

Table S4. Genepy Scores in the Crohn's Chort The table shows the list of genes in the topmost row. Sample IDs in column A have been re-labelled to protect individual information. The column under each gene includes the GenePy scores per individual. Column D shows the sum of the GenePy scores across all the 24 selected genes per individual. Column B shows patients who have had/not had surgery. Column C indicates the periostin levels. The second row includes information on the individual gene length. All the GenePy scores were adjusted for the respective gene length.

Table S5 GenePy scores - UC

Sample ID			Sum Scores	CCR5	CXCR4	CYTIP	EPAS1	IL6	JAG1	LEF1	MMP2	MMP9	NOTCH1	PIK3CG	POSTN	RHOA	ROCK1	SRC	STAT3	TCF7	TCF7L1	TCF7L2	TNC	VCAM1	VEGFA	VHL	WNT2
Gene Lengt	Surgery, no= 1, yes= 2	Periostin (pg/ml)		1044	1247	1681	3202	967	4933	2265	2316	2582	8864	3587	3913	936	5239	2076	3915	2856	2303	3367	7148	2141	2189	872	1200
UC1	1	35092	0.0013426	0	0	0	0	0	9.084E-05	0	0.0001887	0.0003716	0	0.0002243	0.0002784	0	0	4.104E-10	0	0	0	0	5.821E-05	6.641E-06	0.0001239	0	0
UC2	1	25273	0.0010486	0	0	0	0	0	0.000103	0	0.0002378	0.000155	0.0001196	0.0001747	0	0	0	4.104E-10	0	0	0	0	0.0001279	6.641E-06	0.0001239	0	0
UC3	1	31872	0.0005618	0	0	0	0	0	0.0002929	0	0	0.0001552	2.045E-05	5.575E-05	0	0	0	4.104E-10	0	0	0	0	3.093E-05	6.641E-06	0	0	0
UC4	1	35194	0.0015708	0.0006515	0	0	0	0	3.655E-05	0	8.342E-05	0.000155	0.0002299	6.154E-06	0	0	0	4.104E-10	0	0	0	0.0002003	0.0002014	6.641E-06	0	0	0
UC5	1	28386	0.0021867	0.0004292	0	0	0.000244	0	0.0001907	0	0.0004385	0.0003283	3.854E-05	0.0001747	0	0	0	4.104E-10	0	0	0	0	0.0002121	6.641E-06	0.0001239	0	0
UC6	1	15025	0.0018791	0	0	0	0.0001393	0	5.749E-05	0	0.0002378	8.009E-05	6.099E-05	6.154E-06	0	0	0	4.104E-10	0	8.017E-05	0.0001931	0	3.093E-05	6.641E-06	0.0001239	0.0008626	0
UC7	1	16091	0.0013255	0	0	0	0	0	0.0004166	0	0.0004385	0.000155	5.113E-05	6.154E-06	0	0	0	4.104E-10	0	8.017E-05	0	0	0.0001713	6.641E-06	0	0	0
UC8	1	15007	0.0025095	0.0012774	0	0	0.0001393	0	6.765E-05	0	0.0002378	0.0001851	0.0001942	0.0002466	0	0	0	4.104E-10	0	0	0	0	3.093E-05	6.641E-06	0.0001239	0	0
UC9	1	23784	0.0011946	0	0	0	0	0	0.0002529	0	0.0002621	0.0001768	3.854E-05	0.0001636	0	0	0	4.104E-10	0	0	0	0	0.0001702	6.641E-06	0.0001239	0	0
UC10	1	35378	0.001718	0.0004292	0	0	0	0	0.0001184	0	0.0003485	8.009E-05	6.171E-05	0.0002457	0	0	0	4.104E-10	0	0.0001711	0	0	0.0001328	6.641E-06	0.0001239	0	0
UC11	1	24189	0.0009235	0	0	0	0	0	0	0	0.0001077	0.0003283	4.29E-05	0.000114	0	0	0	4.104E-10	0	9.544E-05	0	0	0.0002285	6.641E-06	0	0	0
UC12	1	15327	0.0019201	0	0	0	0	0	0.0002265	0	0.0003485	0.0004395	4.986E-05	0.0002243	0	0	0	4.104E-10	0	0.0003133	0	0	0.0001876	6.641E-06	0.0001239	0	0
UC13	1	32549	0.0017981	0.0004292	0	0	0	0	4.955E-05	0	0	0.0003716	9.637E-05	6.154E-06	0	0	0	4.104E-10	0	0.0001756	0.0004753	0	0.0001876	6.641E-06	0	0	0
UC14	2	31159	0.0035955	0.002436	0	0	0	0	0.0001184	0	0.0004014	8.009E-05	0	6.154E-06	0	0	0	4.104E-10	0	0	0	0	0.0003196	6.641E-06	0.0002272	0	0
UC15	2	36908	0.0016229	0	0	0	0	0	0.0001184	0	0.0005425	0.0001768	5.113E-05	0.000114	9.363E-05	0	0	4.104E-10	0	0.0001497	0	0	0.0001429	6.641E-06	0.0002272	0	0
UC16	1	21654	0.0008582	0	0	0	0	0	6.881E-05	0	0.0001887	0.000155	0.0001287	0.0001961	0	0	0	4.104E-10	0	0	0	0	0.0001143	6.641E-06	0	0	0
UC17	1	26040	0.0016528	0	0	0	0	0.0004105	0	0	0.0004878	8.009E-05	0	0.0002243	0	0	0	4.104E-10	0	8.017E-05	0.0001931	0	0.0001702	6.641E-06	0	0	0
UC18	1	21373	0.0016158	0.0004292	0	0	0	0	0.000107	0	0	0.0003283	4.603E-05	5.575E-05	9.363E-05	0	0	4.104E-10	0	0.0003935	0	0	0.0001557	6.641E-06	0	0	0
UC19	2	25074	0.0009971	0	0	0	0	0	6.881E-05	0	0	0.000155	0.0001109	6.154E-06	0	0	0	4.104E-10	0	0.0002579	0	0.0002003	0.0001914	6.641E-06	0	0	0
UC20	1	35484	0.0006922	0	0	0	0	0	0	0	0.0001887	8.82E-05	1.809E-05	5.575E-05	0	0	0	4.104E-10	0	0.0001756	0	0	0.0001592	6.641E-06	0	0	0
UC21	1	41575	0.0010396	0	0	0	0.0001393	0	0.0001727	0	0.0002378	0.000155	3.068E-05	6.154E-06	0	0	0	4.104E-10	0	9.544E-05	0	0	0.0001958	6.641E-06	0	0	0
UC22	2	25331	0.0013605	0	0	0	0	0	6.881E-05	0	0.0004385	0.0003716	0.0001403	5.575E-05	0	0	0	4.104E-10	0	0	0	0	0.000155	6.641E-06	0.0001239	0	0
UC23	1	27194	0.0016469	0	0	0	0	0	0.0001572	0	0	0.0007957	3.451E-05	0.0002457	0	0	0	4.104E-10	0	0	0	0	0.0001316	0.0001582	0.0001239	0	0
UC24	1	31937	0.0025125	0	0.0007762	0	0	0	0.0002698	0	0.0003772	0.0003283	0.0001182	5.575E-05	0.0002784	0	0	4.104E-10	0	8.017E-05	0	0	0.0002219	6.641E-06	0	0	0
UC25	1	21000	0.0006751	0	0	0	0	0	0.0002929	0	0	8.009E-05	5.496E-05	5.575E-05	0	0	0	4.104E-10	0	0	0	0	0.0001848	6.641E-06	0	0	0
UC26	1	24993	0.0018474	0.0004292	0	0	0	0	0.0001768	0	0.0004385	8.009E-05	0.0001188	0.000114	0	0	0	4.104E-10	0	8.017E-05	0	0	0.0001277	0.0001582	0.0001239	0	0
UC27	1	31252	0.0010659	0	0	0	0	0	0.000107	0	0	0.0003283	8.48E-05	0.0002243	9.363E-05	0	0	4.104E-10	0	0	0	0	6.964E-05	0.0001582	0	0	0
UC28	1	16866	0.0014663	0	0	0	0	0	0.0001042	0	0.0002378	0.0004451	0.0001533	0.0001961	0	0	0	4.104E-10	0	0	0	0	0.0003231	6.641E-06	0	0	0
UC29	2	24901	0.0009364	0	0	0	0	0	0.0001403	0	0.0002721	0.0003716	1.187E-05	6.154E-06	0	0	0	4.104E-10	0	0	0	0	0.0001277	6.641E-06	0	0	0
UC30	1	23482	0.0010529	0	0	0	0	0	0.0001403	0	0	0.000155	5.496E-05	6.154E-06	0	0	0	4.104E-10	0	0	0	0	0.0001279	6.641E-06	0	0	0.0005619
UC31	1	33184	0.0017495	0	0	0	0	0	0.0003381	0	0.0004385	0.0003716	5.496E-05	0.0001636	0	0	0	4.104E-10	0	0.0001939	0	0	5.821E-05	6.641E-06	0.0001239	0	0
UC32	1	22137	0.0021867	0.0004292	0	0	0.000244	0	0.0001907	0	0.0004385	0.0003283	3.854E-05	0.0001747	0	0	0	4.104E-10	0	0	0	0	0.0002121	6.641E-06	0.0001239	0	0
UC33	1	38059	0.0008591	0	0	0	0	0	6.881E-05	0	0.0003485	0.0001768	5.113E-05	6.154E-06	0	0	0	4.104E-10	0	0	0	0	0.0002012	6.641E-06	0	0	0
UC34	2	21480	0.0009923	0	0	0	0	0	0.0001118	0	0	0.0005364	5.496E-05	0.0002243	0	0	0	4.104E-10	0	0	0	0	5.821E-05	6.641E-06	0	0	0
UC35	1	34042	0.0011649	0	0	0	0	0	0.0002495	0	0	0.000418	5.496E-05	8.055E-05	0	0	0	4.104E-10	0	0	0	0.0002003	0.000155	6.641E-06	0	0	0
UC36	1	35992	0.0014193	0	0.0002925	0	0	0	0.0001184	0	0.0002378	0.000155	5.496E-05	0.0002243	0	0	0	4.104E-10	0	0	0	0	0.0002058	6.641E-06	0.0001239	0	0

Table S5. Genepy Scores in the UC Chort

The table shows the list of genes in the topmost row. Sample IDs in column A have been re-labelled to protect individual information. The column under each gene includes the GenePy scores per individual. Column D shows the sum of the GenePy scores across all the 24 selected genes per individual. Column B shows patients who have had/not had surgery. Column C indicates the periostin levels. The second row includes information on the individual gene length. All the GenePy scores were adjusted for the respective gene length.
